# Small-scale fed-batch cultivations of *Vibrio natriegens*: overcoming challenges for early process development

**DOI:** 10.1007/s00449-025-03159-9

**Published:** 2025-04-18

**Authors:** Clara Lüchtrath, Eva Forsten, Romeos Polis, Maximilian Hoffmann, Aylin Sara Genis, Anna-Lena Kuhn, Marcel Hövels, Uwe Deppenmeier, Jørgen Magnus, Jochen Büchs

**Affiliations:** 1 AVT- Biochemical Engineering, RWTH Aachen University, Aachen, Germany; 2 Institute for Microbiology and Biotechnology, University of Bonn, Bonn, Germany

**Keywords:** Fed-batch, Glucose soft sensor, Microtiter plate, *Vibrio natriegens*

## Abstract

*Vibrio natriegens* is a fast-growing microbial workhorse with high potential for biotechnological applications. However, handling the bacterium in batch processes is challenging due to its high overflow metabolism and mixed acid formation under microaerobic conditions. For early process development, technologies enabling small-scale fed-batch cultivation of *V. natriegens* Vmax are needed. In this study, fed-batch cultivations in 96-well microtiter plates were successfully online-monitored for the first time with a µTOM device. Using the online-monitored oxygen transfer rate, a scale up to membrane-based fed-batch shake flasks was performed. The overflow metabolism was efficiently minimized by choosing suitable feed rates, and mixed acid formation was prevented. A glucose soft sensor using the oxygen transfer rate provided accurate estimates of glucose consumption throughout the fermentation, eliminating the need for offline sampling. Analyzing the impact of the inducer IPTG on the recombinant production of the enzyme inulosucrase revealed concentration-dependent effects in batch processes. In contrast, fed-batch operating mode resulted in high inulosucrase activity even without induction. Overall, an inulosucrase titer of 80 U/mL was achieved. In conclusion, the advantages of small-scale fed-batch technologies supported by a glucose soft sensor have been demonstrated for early process development for *V. natriegens* Vmax.

**Supplementary Information:**

The online version contains supplementary material available at 10.1007/s00449-025-03159-9.

## Introduction

The non-pathogenic gram-negative bacterium *Vibrio natriegens* has emerged as a potential new workhorse for biotechnology [1–3] due to its very high substrate uptake- and growth rate, allowing faster processes and higher space–time yields [4]. Since its first isolation over fifty years ago [5], numerous molecular tools have been developed, and research into applications of *V. natriegens* is steadily increasing [6–13]. As a bacterium of marine origin, *V. natriegens* requires high sodium concentrations and tolerates high osmolarities. Due to its high growth rate, strictly sterile conditions are unnecessary, saving time and energy costs [14]. However, like *Escherichia* *coli*, *V. natriegens* exhibits an acetate overflow metabolism at high glucose concentrations [8, 15]. In addition, mixed acid fermentation occurs under anaerobic and micro-aerobic conditions [4, 7] which are easily encountered as the high growth rate leads to high oxygen demand and an increased likelihood of oxygen limitation. Therefore, unregulated processes in early process development are more likely to be pH-inhibited [16]. To meet these challenges and utilize the microorganism’s potential, tools and strategies for working with *V. natriegens* should be explored.

To save time and money, the initial process development is usually carried out in microliter (microtiter plate) to milliliter (shake flask) scale. Over the last decades, engineering parameters in microtiter plates and shake flasks have been extensively researched [17–23] e.g., the oxygen mass transfer [24–30]. In addition, it was shown that cultivations from both microtiter plates and shake flasks can be transferred to fermenters if a suitable scale-up parameter is used, and that there is a good comparability between all scales [31–35].

In large scale, the oxygen transfer rate (OTR) is a common parameter used to quantify the physiological state of aerobic cultures, since most metabolic activities depend on oxygen consumption in stoichiometric ratios [36, 37]. While the OTR is usually monitored by off-gas analysis in large scale, the Respiration Activity Monitoring System (RAMOS) allows the same for shake flasks [36, 38]. It non-invasively monitors the OTR, Carbon Dioxide Transfer Rate (CTR), and the Respiratory Quotient (RQ) in adjustable measurement intervals. In microtiter plates, the recently introduced micro(µ)-scale Transfer Rate Online Measurement device (µTOM) measures the OTR in 96 separate wells [35]. To avoid incomparable cultivation conditions during initial process development that could complicate the subsequent scale-up, small-scale process monitoring is just as important as in large scale [38–40].

Industrial processes predominantly operate under fed-batch conditions [41–43]. Fed-batch processes have the advantage that the feed rate effectively controls the growth rate, as glucose is supplied at a limiting rate [42, 44]. As a result, the oxygen demand is lower than in batch processes, and micro-aerobic conditions, which lead to mixed acid fermentation in *V. natriegens,* are avoided. Moreover, no overflow metabolism occurs [15, 44], and pH shifts become less likely with glucose-limiting conditions [45]. To obtain the most efficient process on an industrial scale, the process development should also be conducted in fed-batch mode [46]. Several technologies are available for small-scale fed-batch applications, ranging from enzymatic systems such as EnBase®/EnPresso® [47], droplet-based feeding systems such as the liquid injection system (LIS, [48] for shake flasks and microfluidic feeding for the BioLector® Pro [41], to diffusion-driven technologies such as FeedBeads® [49] and membrane-based feeding for flasks [50, 51] and FeedPlates® for 48- and 96-well MTPs [42]. To date, few fed-batch processes for *V. natriegens* have been described in the literature [15, 44, 52, 53], and only one study attempted to establish a fed-batch fermentation in small scale using the EnPresso® system [15]. This system enzymatically releases glucose from starch through amylases [47], but is interfered with by intrinsically produced amylases. The diffusion-driven fed-batch systems, in contrast, are not susceptible to amylases [54] and, therefore, a promising alternative. Furthermore, the feasibility of scaling up from diffusion-driven fed-batch shake flasks to stirred tank reactors was previously shown by Müller et al. [55].

For this study, the production of inulin-type fructooligosaccharides (I-FOS) was selected as an application case. In addition to their use in the food industry, I-FOS have gained importance in the pharmaceutical sector due to their health-promoting and functional properties, including antioxidant, anti-inflammatory, anti-tumor and antiviral effects [56, 57]. Generally, I-FOS can be extracted from inulin-rich plant material or synthesized enzymatically [58, 59]. When produced enzymatically, I-FOS can either be formed by the degradation of inulin or directly synthesized using transfructosylating enzymes. The transfructosylating inulosucrase InuGB-V3 from *Lactobacillus gasseri* has been identified as a promising enzyme variant for the production of I-FOS due to its high substrate conversion, which together with low sucrose hydrolysis results in high I-FOS yields [60]. A truncated version of the enzyme, InuGB-V3, has successfully been expressed in *E. coli* [60]. However, the enzymatic process is not yet economically competitive with extraction-based I-FOS production. The expression of InuGB-V3 in another vector in combination with an alternative and fast-growing host, such as *V. natriegens* Vmax, could increase space–time yield and titer and, therefore, be a promising step toward economic viability.

This study demonstrates the early process development of the promising recombinant enzyme InuGB-V3 in the fast-growing host *V. natriegens* Vmax. Due to the high overflow metabolism of *V. natriegens*, a fed-batch process is targeted. This study investigates the suitability of diffusion-driven fed-batch systems for *V. natriegens* Vmax and compares the induction of InuGB-V3 expression in batch and fed-batch mode. So far, diffusion-driven fed-batch in 96-well FeedPlates® has only ever been performed unmonitored. In this study, valuable insights into the process are revealed for the first time through the integration of a µTOM device. Since low cultivation volumes impede regular sampling, the potential of the OTR as a mechanistic soft sensor for glucose consumption will be evaluated. Overall, useful tools and technologies to address challenges of early process development for *V.* *natriegens* Vmax in small scale are presented in this contribution.

## Materials and methods

### Online monitoring techniques for shake flasks and microtiter plates

**Fig. 1:**
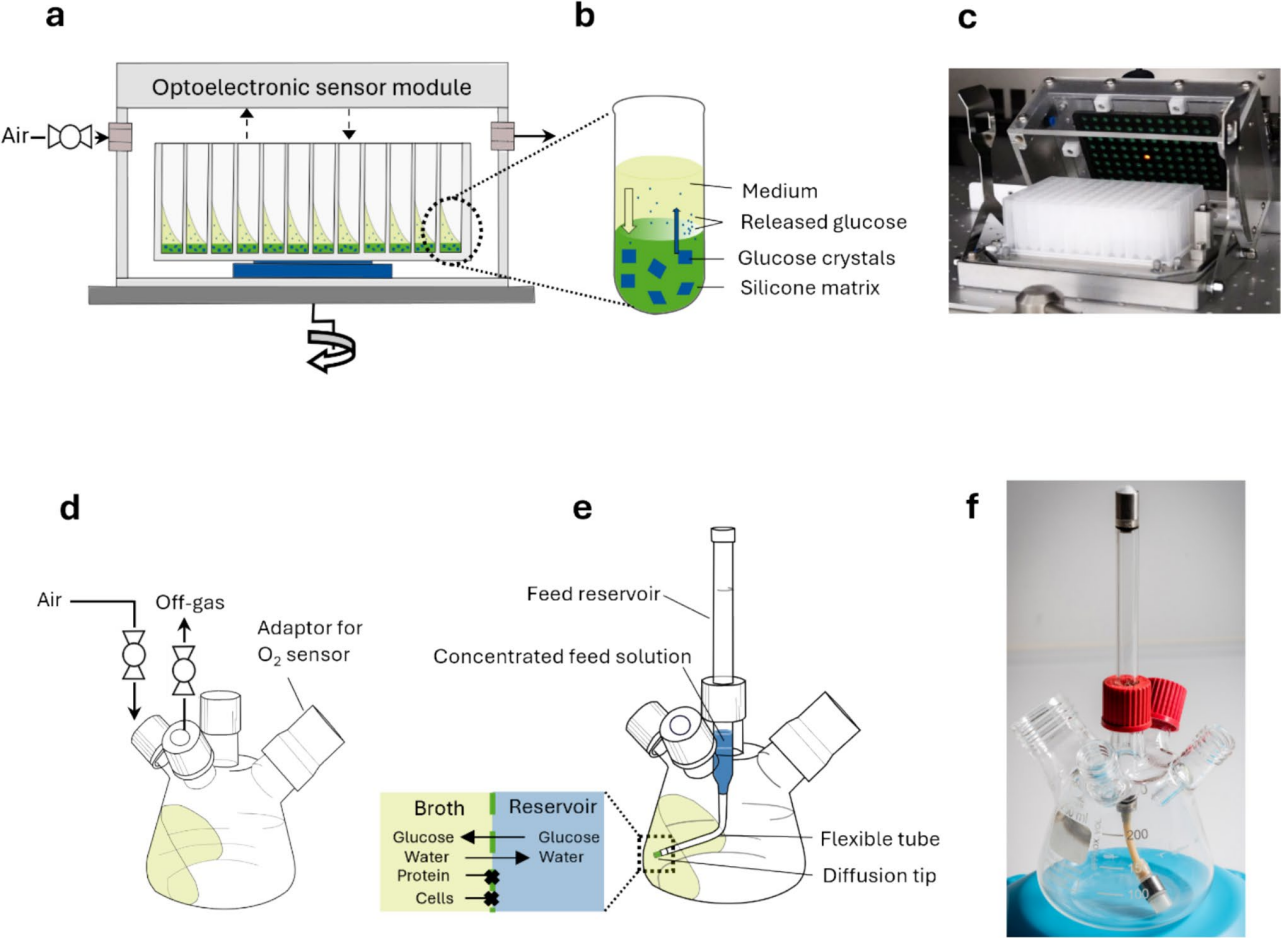
Schematic illustration of the devices used in this work for microtiter plate and shake flask cultivations in fed-batch. **a** µTOM device used to monitor the oxygen transfer rate (OTR) in 96-well microtiter plate experiments (adapted from [35]). **b** Single well of a FeedPlate® (adapted from [61]). Glucose crystals are embedded in a silicone matrix. Upon contact with a liquid medium, water penetrates into the silicone matrix, the glucose crystals gradually dissolve, and the glucose solution is released into the medium. **c** Picture of the µTOM device. **d** Illustration of a RAMOS flask used with a RAMOS device [36] to monitor the oxygen transfer rate. **e** Illustration of a membrane-based fed-batch shake flask (adapted from [55]). The reservoir contains a highly concentrated glucose feed solution, separated from the culture broth by a membrane-covered diffusion tip. A flexible tube allows the tip to rotate with the bulk liquid in the flask. Thus, during shaken cultivations, the membrane-covered diffusion tip is always in contact with the culture. Therefore, glucose is released into the culture broth. Due to osmosis, a weak flow of water enters the feed reservoir in reverse direction to the diffusion of the glucose. Proteins and cells are held back in the culture broth because they cannot pass through the membrane due to the molecular weight cut-off. **f** Picture of the membrane-based fed-batch shake flask

All cultures were monitored non-invasively through their oxygen transfer rate (OTR). For OTR measurements in shake flasks, a custom-built respiration activity monitoring system (RAMOS, Fig. 1d) was used [36, 38]. To determine the OTR in 96-well microtiter plates, a µ-scale transfer rate online measurement device (µTOM, Fig. 1a, c) was used [35].

### Fed-batch technology in microtiter plates and flasks

For fed-batch cultivation in a microtiter scale, FeedPlates® (Fig. 1b) were used, a technology in which glucose crystals are embedded in a silicone matrix at the bottom of each well. When the culture medium comes into contact with the matrix, water diffuses in, dissolves the glucose crystals and glucose is steadily released into the medium [54, 61, 62].

Membrane-based fed-batch shake flasks are derived from the standard RAMOS flasks (Fig. 1d). Because the feed is provided via an adjustable feed solution and reservoir, fed-batch shake flasks allow for more flexible feeding options than FeedPlates®. An advantage is that in addition to glucose or other carbon sources such as glycerol, nitrogen or pH stabilizing agents can be fed to create conditions closer to stirred tank reactors [51, 63]. The feed solution in the feed reservoir is separated from the culture broth through a membrane. The membrane is located at a diffusion tip, which is connected to the feed reservoir through a flexible tube. During shaking, the diffusion tip rotates in phase with the culture broth, keeping the feed reservoir and culture broth constantly in contact. The concentration gradient between the reservoir feed solution and the culture broth drives the diffusive mass transfer across the membrane, and the substrate enters the culture broth. Since the membrane has a molecular weight cut-off of 10 to 20 kDa, proteins and cells cannot pass into the feed solution.

The fed-batch shake flasks, first introduced by Bähr et al. [50], were prepared as Philip et al. [64] and Habicher et al. [63] described. Circular membrane discs (RCT-NatureFlex NP, Reichelt Chemietechnik GmbH + Co., Heidelberg, Germany) of 16 mm diameter were made using a hollow puncher and stretched onto the diffusion tip with an custom-built apparatus. A flexible and biocompatible silicone tube kept the membrane in place. The diffusion tip was filled with 200 µL of deionized water to prevent the membrane from drying. Then, the tip was connected to the reservoir through silicone tubing and sterilized by autoclaving (Fig. S1). The reservoir was filled with 2.8 mL sterile glucose feed solution before cultivation.

### Microorganism

In this study, the truncated version of the inulosucrase InuGB (InuGB-V3) from *Lactobacillus gasseri* DSM 20604 was heterologously produced in *V. natriegens* Vmax X2 (*V. natriegens* ATCC 14048 dns::LacI-T7-RNAP). This organism is from here on referred to as *V. natriegens* Vmax. The expression vector pET19b::inuGB-V3 (Fig. S2) was constructed based on pASK3_InuGB-V3, a vector formerly created by Wienberg et al. [60]. A gene fragment encoding amino acids 37 – 699 of InuGB (Genbank accession: GU166814) was amplified from pASK3_InuGB-V3 using the primers *inuGB-V3_Nco*I_for and *inuGB-V3_Bam*HI_rev (Table 1).

**Table 1:** Primers used for the construction of the expression vector pET-19b::inuGB-V3

Primer	Sequence	Restriction site
*inuGB-V3_Nco*I_for	ATTA**CCATGG**CTACTACTAATGCAG	*Nco*I
*inuGB-V3_Bam*HI_rev	ATTA**GGATCC**CTTTAAGTTATATCCACCAATTAAATCCC	*Bam*HI

Restriction sites are highlighted in bold

The PCR product was purified using NEB’s Monarch® PCR & DNA Cleanup Kit (New England Biolabs, Ipswich, US) and digested using *Nco*I and *Bam*HI. Ligation into the complementary digested pET-19b vector (Merck Millipore, Burlington, US) was achieved by NEB's instant sticky-end ligase master mix, according to the manufacturer's instructions. The ligated vector was transformed into *V. natriegens* Vmax cells (BioCat GmbH, Heidelberg, Germany) by heat shock transformation. Chemically competent cells of *V. natriegens* Vmax were generated using ROTI®Transform (Carl Roth, Karlsruhe, Germany), according to the manufacturer’s instructions. Transformed cells were plated on BHI + v2 agar plates.

The presence of an intact pET19b::inuGB-V3 plasmid in *V. natriegens* Vmax was verified through sequencing. The plasmid was purified from overnight cultures using the “NucleoSpin Plasmid Easy Pure Kit” (Macherey–Nagel, Düren, Germany), following the manufacturer’s instructions and sequenced (Microsynth SeqLab GmbH, Göttingen, Germany). The primer sequence was TAATACGACTCACTATAGGG.

### Media and solutions

Unless stated otherwise, all chemicals were obtained from Carl Roth GmbH + Co. KG (Karlsruhe, Germany).

As recommended by Weinstock et al. [9], Brain Heart Infusion (BHI) supplemented with v2-salts was used for precultures. BHI and v2-salts were autoclaved separately, stored at room temperature, and combined for every preculture. The combined BHI + v2 medium contained 37 g/L Bacto™ Brain–Heart Infusion (Article 237500, Becton Dickinson GmbH, Heidelberg, Germany), 11.9 g/L NaCl, 0.3 g/L KCl, and 4.7 g/L MgCl. For plasmid stability, 100 mg/L carbenicillin from a 100 g/L carbenicillin stock solution (sterile filtered, stored at −20 °C) was added before use.

BHI + v2 agar plates contained the ingredients described above and 15 g/L agar–agar (Kobe I), as well as 50 mg/L ampicillin for plasmid selection.

Main cultures were conducted in a modified Wilms-MOPS medium [65–67]. It consisted of the following stock solutions: 500 g/L glucose solution, 4 × main salts solution containing 27.92 g/L (NH_4_)_2_SO_4_, 12 g/L K_2_HPO_4_, and 8 g/L Na_2_SO_4_ (set to pH 7.5 with NaOH); 5 × 3-(*N*-Morpholino)-propane sulfonic acid (MOPS) stock solution containing 418.5 g/L MOPS (equals 2 M MOPS, set to pH 7.5 with NaOH); 26.67 × NaCl solution containing 200 g/L NaCl; 100 × MgSO_4_ solution containing 50 g/L MgSO_4_ × 7 H_2_O; 1000 × thiamin containing 10 g/L thiamin-HCl. The 1000 × carbenicillin stock contained 100 g/L carbenicillin. The 1000 × trace element solution contained 0.54 g/L ZnSO_4_ × 7 H_2_O (Merck KGaA, Darmstadt, Germany), 0.48 g/L CuSO_4_ × 5 H_2_O (Merck KGaA, Darmstadt, Germany), 0.3 g/L MnSO_4_ x H_2_O, 41.76 g/L FeCl_3_ × 6 H_2_O, 1.98 g/L CaCl_2_ × 2 H_2_O, 33.4 g/L Na_2_EDTA × 2 H_2_O (Merck KGaA, Darmstadt, Germany), and 0.54 g/L CoCl_2_ × 6 H_2_O. The main salt-, NaCl-, glucose, and MgSO_4_ stock solutions were autoclaved and stored at room temperature. The MOPS buffer solution was sterile-filtered and stored at room temperature. The thiamin, carbenicillin, and trace element solutions were sterile-filtered and stored at 4°C (the latter protected from light). The medium was freshly prepared for each cultivation by combining the stock solutions according to their concentration factor, adding the desired amount of glucose and inoculum, and filling the remaining volume with sterile deionized water.

### Precultures

Per RAMOS flask, 8 mL of BHI + v2 medium was inoculated to an initial optical density (OD_600_) of 0.05 from a cryo culture. The flask was then incubated at 37 °C on an orbital shaker (ISF1-X, Kühner AG, Birsfelden, Switzerland). The shaking frequency was set to 350 rpm at a shaking diameter of 50 mm. Once the preculture reached the late exponential growth phase after 3.5–4 h, it was used to inoculate the main culture.

### Main culture in batch and fed-batch

For shake flask cultivations, 250 mL RAMOS flasks were filled with either 8 mL of inoculated medium (initial OD_600_ 0.5) for batch cultivations or 10 mL for fed-batch cultivations and incubated on an orbital shaker (ISF1-X, Kühner AG, Birsfelden, Switzerland) at 350 rpm shaking frequency and a shaking diameter of 50 mm. The cultivation temperature was set to 30 °C unless stated otherwise. RAMOS flasks were connected to a custom-built RAMOS device [36, 38] using the following measurement settings: 10 min low flow phase, 4 min measurement phase, 1 min high-flow phase.

For microtiter plate cultivations, either 96-well DeepWell plates (J.T.Baker®, Plate Medio (2 mL), VWR International GmbH, Darmstadt, Germany) or FeedPlates® (SMFP04001, Kuhner shaker, Herzogenrath, Germany) were filled with 100 or 200 µL inoculated medium (initial OD_600_ 0.5) per well. The microtiter plate was mounted on an orbital shaker in a µTOM device [35] and monitored using the following measurement settings: 10 min low flow phase and 5 min measurement phase. The shaker and incubation hood were set to 37 °C, 80% humidity, and 1000 rpm shaking frequency at 3 mm shaking diameter.

For some of the FeedPlate® cultivations, a washing step was conducted prior to cultivation. Each well was filled with 1 mL of sterile water and the plate incubated at room temperature for 24 h. After this, the water was discarded.

### Offline analysis

A calibrated pH meter (HI 221, Hanna Instruments, Germany) was used for the pH measurements. OD_600_ measurements at 600 nm were performed using a Genesys 20 photometer (Thermo Scientific, Dreieich, Germany). The culture broth was diluted to OD_600_ 0.1—0.3 [68, 69] with 9 g/L NaCl.

HPLC samples were centrifuged, and the supernatant was filtered using either 96-well filter plates (AcroPrep™ Advance 96 Filter Plate 0.2 µm Supor, Pall Life Sciences, Dreieich, Germany) for microtiter plate experiments or syringe filters (Rotilabo syringe filter cellulose acetate 0.2 µm, Carl Roth GmbH + Co. KG, Karlsruhe, Germany) for shake flask experiments. Glucose and acetate concentrations were determined using an organic acid column (300 × 8 mm, ROA-Organic Acid H+ , Phenomenex, Aschaffenburg, Germany) in combination with a Prominence high-performance liquid chromatography (HPLC, Shimadzu Europe, Düsseldorf, Germany) with a refractive index detector at the following settings: temperature 75 °C, carrier flow 0.8 mL/min 0.25 mM sulfuric acid.

### Endpoint determination of the feed rate in membrane-based fed-batch shake flasks

Prior to each fed-batch cultivation, the shake flask and feed reservoirs were weighed separately on a scale (LA 254, VWR International GmbH, Darmstadt, Germany, Fig. S1). Weighing was repeated immediately once the reservoir was filled with feed solution and the flask filled with culture broth. After stopping the cultivation, each flask and respective reservoir were weighed separately. The residual glucose concentration of the feed solution in the reservoir was determined via HPLC at the end of the cultivation. The filling volume of the feed solution in the feed reservoir was then calculated using the solution's density and weight. From the volume and concentration, the final amount of glucose in the feed reservoir was calculated. The total glucose consumption was determined by subtracting the final amount from the initially supplied glucose. Finally, the feed rate [g/L/h] was calculated by division through the volume of the culture broth and the cultivation time.

### Cell lysis and determination of inulosucrase activity

First, samples were centrifuged for 10 min (room temperature, 18,000 g), and the supernatant was discarded. Then, the pellets were frozen at −20 °C overnight to facilitate extraction. After thawing, cell lysis was performed using BugBuster® (Merck, KGaA, Darmstadt, Germany), following the manufacturer’s instructions with the addition of Benzonase® (Sigma Aldrich, Steinheim, Germany) and lysozyme (Carl Roth, Karlsruhe, Germany). The remaining cell extract was mixed 1:1 with 800 g/L glycerol and stored at −20 °C.

One unit (U) of inulosucrase activity is defined as the release of 1 µmol of glucose per minute from the substrate sucrose. In the inulosucrase-mediated reaction from sucrose to inulin-type fructooligosaccharides and glucose, the amount of glucose equals the amount of sucrose used as a fructose donor in the reaction. The recently introduced Real-time GOPOD assay [70] exploits this relationship and quantifies the inulosucrase activity by measuring the glucose release over time. Commercially available GOPOD reagent (Megazyme Ltd., Bray, Ireland) was modified to a so-called Real-time GOPOD reagent, as described by Ehinger et al. [70]. For the Real-time GOPOD assay, 74.4 µL Real-time GOPOD reagent, 100 µL 2 M sucrose in 50 mM Bis–Tris buffer (AppliChem, Darmstadt, Germany), and 5.6 µL deionized water were combined in each well of a 96-well MTP (Carl Roth, Karlsruhe, Germany). The MTP was placed in a plate reader at 30 °C (Synergy MX Reader, Thermo Fisher Scientific, Massachusetts, USA), and the baseline absorbance was monitored at 510 nm for 10 min until the linear reaction phase was reached. To start the inulosucrase reaction, 20 µL of the sample was added to each well, and the absorbance was measured at 510 nm and 30 °C for 30 min. The slope of the linear increase for the reaction with and without a sample was determined. After that, the enzyme activity was calculated by Eq. (1) and Eq. (2):

1 $$\Delta A \left[\frac{1}{min}\right]= \frac{{slope}_{w/_{sample}}\left[\frac{1}{d}\right]-{slope}_{w/o_{sample}}\left[\frac{1}{d}\right] }{1440 [\frac{min}{d}]}$$

2 $$Activity \left[\frac{U}{mL}\right]= \frac{\Delta A [\frac{1}{min}]}{{F}_{cal.}[\frac{L}{g}]}\times\frac{{V}_{well} [L]}{{M}_{glc} [\frac{g}{\mu mol}]}\times\frac{1}{{V}_{sample}[mL]}\times{DF}_{assay}\times{DF}_{sample}$$

With:

$\Delta A$ $\left[\frac{1}{min}\right]$= difference between slopes of the baseline and sample-added reaction.

${F}_{cal.}$ $\left[\frac{L}{g}\right]$ = calibration factor (1.92) correlating the absorption to the glucose concentration.

${V}_{well}$ $[L]$ = volume of the assay solution in each well.

${M}_{glc}$ $\left[\frac{g}{\mu mol}\right]$ = molar mass of glucose.

${V}_{sample}[mL]$ = volume of sample added to each well during the assay.

${DF}_{assay}$ = dilution of the sample due to the assay protocol.

${DF}_{sample}$ = pre-dilution factor of the sample.

For each assay, samples were tested in at least two dilution steps, each with at least two technical replicates. As a standard, and to account for variations between reagent batches, an 80 U/mL biological reference was tested on each assay plate. All measured activities were normalized to this standard according to Eq. (3).

3 $${Activity}_{normalized}\left[\frac{U}{mL}\right]={Activity}_{sample}\left[\frac{U}{mL}\right] \times \frac{80\frac{U}{mL} }{{Activity}_{standard} \left[\frac{U}{mL}\right]}$$

### Calculations

The stoichiometry for aerobic growth of *V. natriegens* with the biomass composition C_1_H_x_O_y_N_z_ on Wilms-MOPS medium is described by Eq. (4). Wilms-MOPS medium contains glucose as the sole carbon source and ammonia as the sole nitrogen source.

4 $${\nu }_{glucose} {C}_{6}{H}_{12}{O}_{6}+ {\nu }_{{NH}_{3}} {NH}_{3}+ {\nu }_{{O}_{2}} {O}_{2} \to {\nu }_{biomass} C{H}_{x}{O}_{y}{N}_{z}+ {\nu }_{{CO}_{2}} {CO}_{2}+ {\nu }_{{H}_{2}O} {H}_{2}O$$

The stoichiometric coefficient ${\nu }_{biomass}$ calculates as described in Eq. (5), given that the yield coefficient ${Y}_{x/s}$ and the coefficients x, y, and z of the biomass composition are known.

5 $${\nu }_{biomass}={Y}_{x/s}\left[\frac{g}{g}\right]\times \frac{{M}_{glc}\left[\frac{g}{mol}\right]}{{M}_{biomass}\left[\frac{g}{mol}\right]}$$

With:

${\nu }_{biomass}$ = the stoichiometric coefficient.

${Y}_{x/s} \left[\frac{g}{g}\right]$ = the yield coefficient.

${M}_{glc} \left[\frac{g}{mol}\right]$ = the molar mass of glucose.

${M}_{biomass} \left[\frac{g}{mol}\right]$ = the molar mass of biomass.

Equation (4) is solved using the stoichiometric coefficient ${\nu }_{biomass}$ from Eq. (5). To solve the equation, the stochiometric coefficient ${\nu }_{glucose}$ is set to 1. Equations (6)–(9) describe the stoichiometric coefficients ${\nu }_{{NH}_{3}}$, ${\nu }_{{H}_{2}O}$, ${\nu }_{{CO}_{2}}$ and ${\nu }_{{O}_{2}}$.

6 $${\nu }_{{NH}_{3}}={\nu }_{biomass}\times z$$

7 $${\nu }_{{H}_{2}O}=\frac{1}{2}(12+ {\nu }_{{NH}_{3}}\times 3-{\nu }_{biomass}\times x)$$

8 $${\nu }_{{CO}_{2}}=6-{\nu }_{biomass}$$

9 $${\nu }_{{O}_{2}}=\frac{1}{2}({\nu }_{biomass}\times y + {\nu }_{{CO}_{2}}\times 2+{\nu }_{{H}_{2}O}-6)$$

With Eq. (4) and the known stoichiometric coefficient for oxygen and glucose, the relationship between oxygen and glucose consumption during aerobic growth can be expressed as the stoichiometric ratio $A \left[\frac{{mmol}_{{O}_{2}}}{{g}_{glucose}}\right]$ (Eq. 10).

10 $$A \left[\frac{{mmol}_{{O}_{2}}}{{g}_{glucose}}\right]=1000 \frac{mmol}{mol}\times {\nu }_{{O}_{2}}\times \frac{{\nu }_{glucose}}{{M}_{glc} \left[\frac{{g}_{glucose}}{mol}\right]}$$

## Results and discussion

### Glucose consumption soft sensor based on the oxygen transfer rate

Since initial process development is often performed in microtiter plates, only a few hundred microliters are available, making intensive offline analysis challenging. To reduce the necessary sampling volume, we decided to apply a glucose soft sensor. The relationship between oxygen consumption and glucose metabolism was described in stoichiometric terms for aerobic conditions in the Calculations section, and monitoring glucose consumption through the oxygen transfer rate (OTR) was targeted.

As a first step, calculations were performed to estimate the expected order of magnitude and the dependence on fluctuations of the empirical values. As described in the Calculations section, the input values were the sum formula of the biomass and the yield coefficient ${Y}_{x/s}$. So far, only a single sum formula for the biomass of *V. natriegens* has been published [71]. In this work, a second sum formula was calculated from the supplemental data of Long et al. [72], where no direct sum formula was presented. Regarding the yield coefficient, the calculation is exemplarily performed with ${Y}_{x/s}$ = 0.44 from Long et al. [72], who determined the coefficient in a batch cultivation. In addition, a value of ${Y}_{x/s}$ = 0.49, determined in fed-batch by Schulze et al. [73], and a value of ${Y}_{x/s}$ = 0.54, from own experimental fed-batch data, were used for calculations. Literature yield coefficients for *V. natriegens* on glucose vary. This is due to the yield coefficient usually being determined empirically and is, thus, subject to a double measurement error: an error of the biomass and an error of the substrate consumption. The calculation was performed for both sum formulas and yield coefficients, which resulted in six different theoretical values for factor $A \left[\frac{{\text{mmol}}_{{\text{O}}_{2}}}{{\text{g}}_{\text{glucose}}}\right]$**. **Table 2 shows these results.

**Table 2:** Estimated range of the stoichiometric ratio $\text{A }\left[\frac{ {\text{mmol}}_{{\text{O}}_{2}}}{{\text{g}}_{\text{glucose}}}\right]$ calculated from literature and own experimental input values

Biomass composition		Batch $\text{A }\left[\frac{{\text{mmol}}_{{\text{O}}_{2}}}{{\text{g}}_{\text{glucose}}}\right]$ for Y_xs_ = 0.44, Long et al. [72]	Fed-batch $\text{A }\left[\frac{{\text{mmol}}_{{\text{O}}_{2}}}{{\text{g}}_{\text{glucose}}}\right]$ for Y_xs_ = 0.49, Schulze et al. [73]	Fed-batch $\text{A }\left[\frac{{\text{mmol}}_{{\text{O}}_{2}}}{{\text{g}}_{\text{glucose}}}\right]$ for Y_xs_ = 0.54, own data
Erian et al. [71]	C_1_H_1.77_O_0.61_N_0.153_ (*V. natriegens*)	15.40	13.37	11.18
Long et al. [72]	C_1_H_1.73_O_0.58_N_0.27_ (*V. natriegens*)	17.18	15.34	13.37

Overall, the calculated values for the stoichiometric ratio *A* range from 11.18 to 17.18 $\frac{{\text{mmol}}_{{\text{O}}_{2}}}{{\text{g}}_{\text{glucose}}}$. The lowest values were obtained from the Erian et al. [71] biomass composition and the highest from Long et al. [72]. Compared to the batch yield coefficient results, the two yield coefficients obtained in the fed-batch process give lower values for the stoichiometric ratio *A* $\left[\frac{{\text{mmol}}_{{\text{O}}_{2}}}{{\text{g}}_{\text{glucose}}}\right]$. The key information is that the stoichiometric ratio *A* is subject to strong fluctuations at small changes in the input values and, therefore, should be determined empirically for further use. Nonetheless, the order of magnitude can already be narrowed down and is expected to fall between 10 and 18 $\frac{{\text{mmol}}_{{\text{O}}_{2}}}{{\text{g}}_{\text{glucose}}}$.

**Fig. 2:**
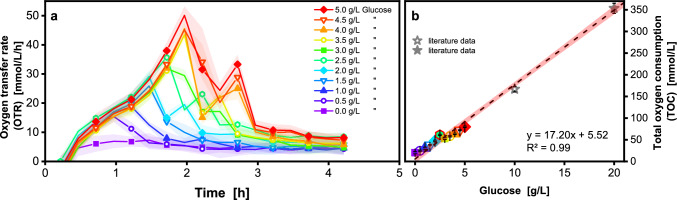
Calibrating the oxygen transfer to glucose consumption in a 96-deep well plate in a batch cultivation. Non-induced batch cultivation of *V. natriegens* Vmax pET19b::inuGB-V3 in modified Wilms-MOPS medium (0–5 g/L glucose) with 400 mM MOPS buffer. Initial OD_600_ 0.5, 37 °C, 1000 rpm at 3 mm shaking diameter, oxygen transfer rate monitored using a µTOM device. **a** 100 µL filling volume in a 96-deep well plate. For clarity, only every third data point is shown as a symbol. Shadows indicate standard deviation for n = 4 replicates. **b** Linear correlation of the total oxygen consumption (shown in Fig. S3a) to the provided and total consumed glucose. The stars represent online data from microtiter plate cultivations from Forsten et al. [67]. Error bars are hardly visible, as they are mostly within the size of the symbols (Fig. S3b). 95% confidence band of the linear fit (dashed line) is shown as a red shadow

For an empirical measurement of the stoichiometric ratio *A* $\left[\frac{{\text{mmol}}_{{\text{O}}_{2}}}{{\text{g}}_{\text{glucose}}}\right]$, batch cultivations with varying glucose concentrations from 0 to 5 g/L were performed using a filling volume of 100 µL (Fig. 2a). The OTR was monitored at 15 min intervals. Figure 2a shows an increasing OTR for all glucose-containing cultures. After glucose consumption, the respiratory activity declines as indicated by a drop in the OTR. As it takes more time to consume more glucose, OTR peaks occur later at higher glucose concentrations. At glucose concentrations > 2 g/L, a second OTR peak becomes apparent. This peak marks the consumption of the overflow metabolite acetate, which *V. natriegens* produces at excess glucose concentrations [36, 38, 72]. In a master mix plate sampled 10 min after the start of cultivation, HPLC analysis showed a higher concentration of acetate for a higher initial concentration of glucose supplied, in good agreement with the OTR observations (Table S1). It is noteworthy that even cultures without glucose show baseline respiratory activity. Since the preculture was carried out in complex medium, complex components may have been carried over to the main culture and consumed by *V. natriegens*.

With higher filling volumes of 200 µL (Fig. S4) a constant OTR is visible for cultivations with > 1.5 g/L glucose. This results from an oxygen limitation [36] which is prolonged with higher glucose concentrations. To prevent mixed acid formation and unwanted pH shifts [16] oxygen-limited cultivation conditions should be avoided by lowering the filling volume or introducing a fed-batch process. While a lower filling volume increases the maximum oxygen transfer capacity (OTR_max_) in shaken bioreactors [30, 35, 74], an unnecessary low filling volume results in elevated levels of water evaporation. To define the optimal filling volume required to circumvent oxygen-limited conditions, it is necessary to determine the OTR_max_. For 96-deep well plates with round well and U-shaped bottom geometry, only a single equation is available in the literature for the calculation of the OTR_max_ for growth on a complex medium [35]. Depending on the expected OTR of the culture and the medium osmolality, the optimal filling volume has to be determined experimentally, as shown in Fig. S5. An OTR_max_ > 50 mmol/L/h was found for a filling volume of 100 µL under the same shaking conditions. For the low glucose batch as well as fed-batch conditions explored in this study, oxygen transfer is not limiting.

For the empirical determination of the stoichiometric ratio *A* $\left[\frac{{\text{mmol}}_{{\text{O}}_{2}}}{{\text{g}}_{\text{glucose}}}\right]$, the total oxygen consumption (TOC) was derived by integrating the OTR (Fig. S3). Higher glucose concentration correlated linearly with a higher TOC (Fig. 2b) as well as the final OD_600_ measured after the cultivation (Fig. S3). A linear fit (Eq. 11) was determined by plotting the TOC against the respective glucose concentration (Fig. 2b). Since the linear fit should be applied as a glucose soft sensor for fed-batch fermentations, where higher glucose concentrations are fed during cultivation, TOC data from literature with higher glucose concentrations was included to ensure an appropriate calibration range. The literature values were obtained through non-oxygen limited microtiter plate cultivations [67].

11 $$y=A*x+b$$

The y-axis intercept *b* reflects the baseline respiratory activity due to the transfer of complex components from the preculture. For oxygen-unlimited conditions, a stoichiometric ratio *A* of 17.20 $\frac{{\text{mmol}}_{{\text{O}}_{2}}}{{\text{g}}_{\text{glucose}}}$ was determined. The value for *A* is in the upper range of the previously calculated theoretical values. The linear fit should be considered as a simplified correlation. The correlation only considers growth on glucose under aerobic conditions and not, for example, under anaerobic conditions. Figure 2a shows that under batch conditions, *V. natriegens* experiences an excess of glucose and consequently converts up to 25 % of the carbon flux to acetate [8, 15], which leads to a pH drop if the system is not sufficiently buffered.

### Establishing a small-scale fed-batch process for Vibrio natriegens

**Fig. 3:**
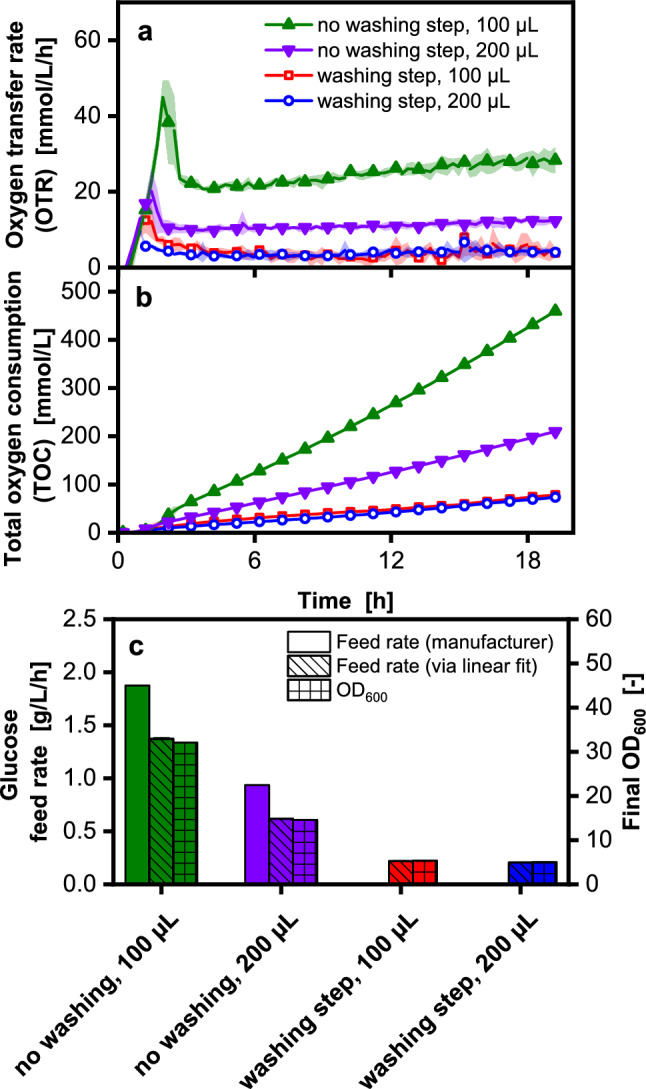
Application of the calibration from Fig. 2c, d to determine the glucose consumption in a fed-batch cultivation in microtiter plates. Non-induced fed-batch cultivation of *V. natriegens* Vmax pET19b::inuGB-V3 in modified Wilms-MOPS medium (no additional initial glucose) with 400 mM MOPS buffer. Initial OD_600_ 0.5, 37 °C, 1000 rpm at 3 mm shaking diameter. The oxygen transfer rate was monitored using a µTOM device. 100 µL or 200 µL filling volume in 96-well FeedPlate® (SMFP04001, high release). For washing step see chapter Main culture in batch and fed-batch. **a** Oxygen transfer rate over time. **b** Total oxygen consumption over time. **c** Comparison of glucose consumption determined from the total oxygen consumption (hatched bars) after 18.7 h (see Fig. 2b), technical data from the manufacturer (empty bars) and final OD_600_ (dotted bars). For clarity, only every third data point is shown as a symbol. Shadows indicate minimum/maximum for n = 2 replicates

The easiest way to avoid conditions with excess glucose is by cultivating in fed-batch mode. During a fed-batch process, glucose is only fed at a rate that is lower than the maximum consumption rate of the organism [42, 44, 75]. Therefore, small scale fed-batch fermentations were conducted in MTPs. Furthermore, the generated fit from Fig. 2b was applied to these cultivations to evaluate their applicability as soft sensors in fed-batch fermentations. To avoid overflow metabolism in a fed-batch process, the glucose feeding rate must be below the critical rate at which the metabolism starts to form acetate. A straightforward way to find a suitable feed rate fast is to carry out FeedPlate® cultivations [61] (Fig. 3). The feed rate was varied by a) variation of the filling volume and b) application of a washing step to lower the initial glucose release (see Main culture in batch and fed-batch in the Material and Methods section) [54]. The combined variation of both methods aimed to generate four different feed rates in the same MTP for a fast screening of suitable feed rates. These initial experiments aimed to validate the soft sensor, conduct a preliminary investigation into the growth of *V. natriegens* Vmax in fed-batch, and assess whether overflow metabolism occurs in *V. natriegens* in the range of feeding rates tested. The OTR curves in Fig. 3a show an initial increase, cumulating in a peak after 2–4 h. During this time, the glucose release of the FeedPlate® is higher than the consumption rate of the organism. Therefore, glucose accumulates, and the culture enters a batch phase [45]. After the batch peak, the OTR decreases to a plateau. This plateau indicates the fed-batch phase, where the glucose release is below the organisms' maximum consumption capacity and carbon-limiting conditions are present [61]. Since the total glucose release per well is constant for the washed and non-washed wells, cultures with a filling volume of 100 µL experience a higher volumetric feeding rate than those with 200 µL filling volume. As the oxygen uptake of the cultures correlates to their glucose uptake, higher OTR values are obtained for the fed-batch cultivations with lower filling volume [54].

When comparing the OTR of cultures after a washing step to cultures without a washing step, a lower batch peak and a shorter batch phase are observed (Fig. 3a). Washing with water avoids a burst of glucose release at the beginning of the batch phase, as the glucose initially released in large quantities is removed during this step. Consequently, less glucose is accumulated during the batch process [54]. Glucose release is also reduced during the fed-batch phase, when a washing step is introduced, which becomes visible as a lower OTR plateau around 4 mmol/L/h. The highest TOC (Fig. 3b) is achieved with the lowest filling volume and without a washing step. None of the cultures showed a second OTR peak during the batch phase. This indicates that overflow metabolism was avoided at all four feeding rates (see Fig. 3c), since no acetate (0.0 g/L) was HPLC-detected after the cultivation.

To evaluate the suitability of the OTR as a glucose soft sensor, the feed rate was calculated using the TOC from Fig. 3b and the linear fit from Fig. 2b. The calculated feed rate for non-washed cultures was 1.37 g/L/h for 100 µL cultures and 0.61 g/L/h for 200 µL cultures (Fig. 3c). The FeedPlate® manufacturer specifies the glucose release at 1.88 g/L/h for 100 µL and 0.94 g/L/h for 200 µL. Since for higher feed rates, more biomass was expected, the final OD_600_ was measured and found to correlate well with the calculated feed rates. Thus, the OTR and linear fit can be combined into a soft sensor for glucose consumption.

To avoid oxygen limitation (Fig. S3) and achieve high feed rates (Fig. 3), only small filling volumes could be used for *V. natriegens* cultivations in 96-well microtiter plates. Since extensive offline sampling for OD_600_, pH, HPLC analysis, and enzyme activity determination was to be performed at the end of each cultivation, the process was scaled up to flask scale. The chosen scale-up criterion was a constant OTR during the fed-batch phase.

**Fig. 4:**
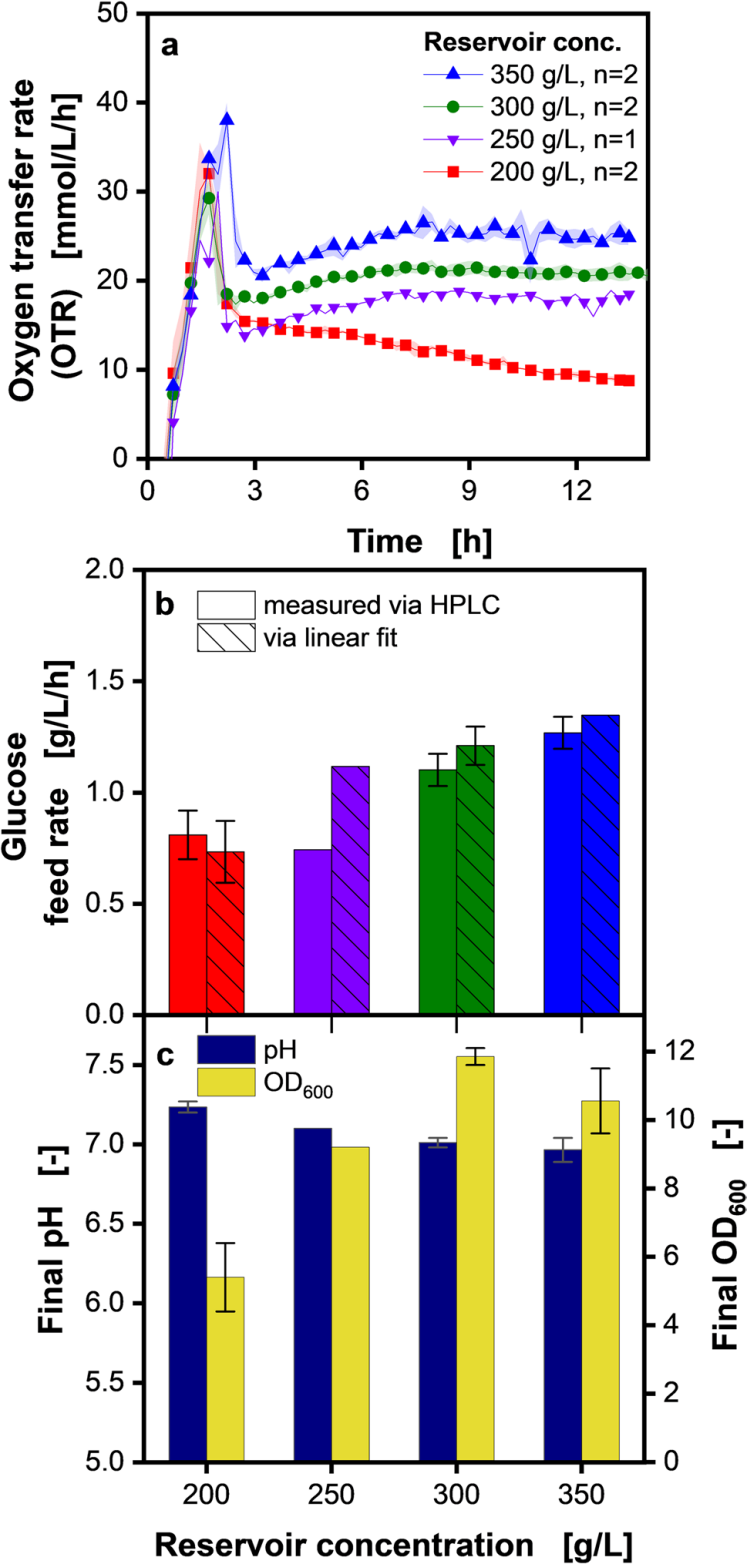
Membrane-based fed-batch cultivation in shake flasks with different glucose feeding rates. Non-induced fed-batch cultivation of *V. natriegens* Vmax pET19b::inuGB-V3 in modified Wilms-MOPS medium (no initial glucose) with 400 mM MOPS buffer. 10 mL filling volume in 250 mL RAMOS flasks, initial OD_600_ 0.5, 37 °C, 350 rpm at 50 mm shaking diameter, the oxygen transfer rate was monitored using a RAMOS device. **a** Oxygen transfer rate over time for different glucose concentrations in the reservoir. For clarity, only every third data point is shown as a symbol. Shadows indicate minimum/maximum for n = 2 biological replicates. The replicate cultivations were conducted independently on different days. **b** Glucose feed rates corresponding to different reservoir concentrations: either the feed rate was offline measured through HPLC analysis (open bars) or determined via the linear fit (hatched bars) presented in Fig. 2b. Error bars indicate minimum/maximum for n = 2 biological replicates. (c) Offline determined final pH and OD_600_

In membrane-based fed-batch shake flasks, the feed rate and, consequently, the OTR level in the feed plateau is determined by the glucose concentration in the central reservoir [50]. To determine the feed rate, corresponding to the previous FeedPlate® cultivation, the glucose concentration in the feed reservoir of the shake flask was varied between 200 and 350 g/L (Fig. 4). The respiration activity revealed an initial OTR increase of all cultures over the first 2.5 h, that ended in an OTR peak at the end of the batch phase (see discussion of Fig. 3). Afterwards, all cultures with a feed reservoir concentration > 200 g/L exhibit a constant OTR which indicates a fed-batch phase. Generally, a higher OTR is observed with increasing concentration of the feed reservoir. More glucose diffuses across the membrane at higher glucose concentrations and is available to the organisms. This is due to the higher concentration gradient between the feed solution and the culture broth [50, 51].Neither residual glucose nor acetate was detected (0.0 g/L glucose and 0.0 g/L acetate) via HPLC after any of the cultivations shown in Fig. 4. Only for a fed-batch performed with 500 g/L glucose in the feed reservoir (Fig. S6), acetate was measured in the culture broth (0.8 ± 0.1 g/L).

The OTR slowly decreases for the two biological replicates with a 200 g/L feed reservoir concentration instead of forming a plateau. The endpoint OD_600_ of 5.8 indicates that biomass was formed and consequently, glucose was fed and fully taken up (no residual glucose HPLC-detected in endpoint samples). Since the same fed-batch conditions had resulted in the formation of an OTR plateau in *B. licheniformis* [63], an issue with the membrane-based fed-batch technology was ruled out. To our knowledge, the observation of low respiratory activity at too low glucose feeding rates has not been reported in the literature so far. The closest phenomenon described in the literature is a decrease in cell dry weight when switching from exponential glucose to constant glucose feeding [15]. The cause remains unclear and should be investigated in future works.

The assumption that more glucose was fed into the shake flask at higher feed reservoir concentrations is validated in Fig. 4b. The feed rate was evaluated offline through HPLC measurement of the feed reservoir solution (see section Endpoint determination of the feed rate in membrane-based fed-batch shake flasks) and compared to the feed rate derived through the OTR soft sensor. In theory, the feed rate should be directly proportional to the feed reservoir concentration since the latter determines the diffusion rate. The offline measurement values display an increasing trend with increasing reservoir concentration, except for the cultivation with a reservoir concentration of 250 g/L. Both methods, the HPLC and soft sensor method, adequately quantify the release of glucose. However, the offline method is quite labor-intensive: Due to diffusion of water back into the feed reservoir because of osmotic effects, diluting effects have to be accounted for by determining the reservoir filling volume at the end of the cultivation, in comparison to the beginning of the cultivation. This is done by weighing the shake flasks before and after the cultivation [63] (Fig. S1). The shake flask is weighed empty and (1) without the reservoir, (2) with the reservoir, (3) with a filled reservoir and (4) with a filled flask. After the cultivation, the flask is first weighed with the reservoir and afterwards weighed without. The initial and final concentration of the feeding solution in the reservoir is then determined via HPLC, which takes several hours. In contrast, the soft sensor method, based on the evaluation of the total oxygen consumption (Fig. 2b), allows to obtain the glucose release rate without delay. At the same time, this method reduces the manual handling steps. In addition, real-time estimation of glucose consumption at any point of the cultivation is possible. Due to these advantages, the soft-sensor is applied for all further shake flask experiments.

Apart from the cultivation with 350 g/L glucose, the final OD_600_ (Fig. 4c) comparison shows an OD_600_ increase with increasing glucose concentration. Higher feed rates give the organisms more glucose, resulting in more biomass. The pH shows an inverse trend. The pH decreases as more ammonia is taken up [15], with a linear relationship between the pH decrease and the feed rate increase. No overflow metabolites were detected at the end of the cultivations (data not shown). Since the OTR plateau of the FeedPlate® matches the OTR plateau of the shake flask cultivation with a 300 g/L glucose feed solution (Fig. S7), this feed reservoir concentration was used for further process development.

According to the literature, the optimal growth temperature for *V. natriegens* is 37 °C [5]. Since protein misfolding is more likely to occur at high temperatures, the temperature for protein expression is often lowered to increase the yield of active protein [76–78]. However, not only protein folding is affected by temperature, but glucose diffusion as well [79, 80]. At 30 °C, the feed rate was determined to be almost as high as at 37 °C (Fig. S8).

### Development of an inulosucrase production process

**Fig. 5:**
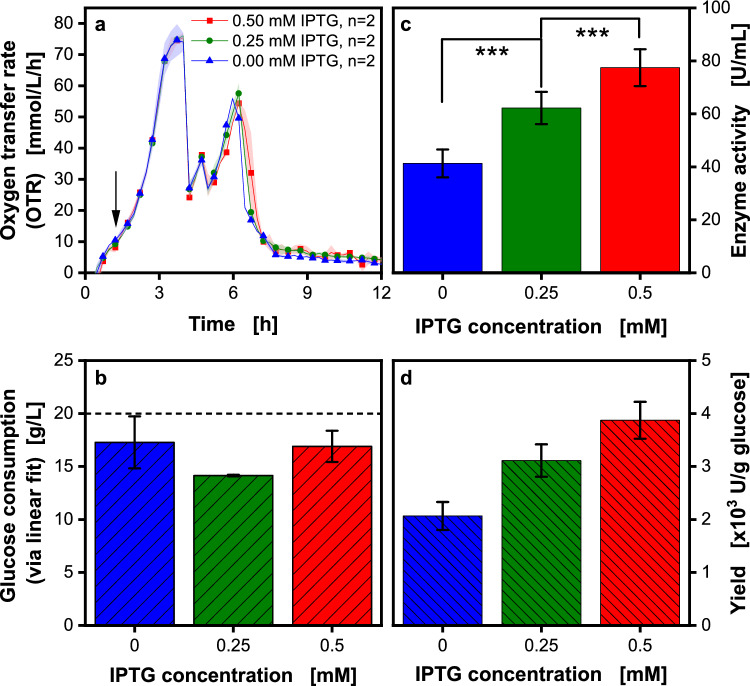
Influence of the inducer concentration (IPTG) on inulosucrase production in a batch process in shake flasks. *V. natriegens* Vmax pET19b::inuGB-V3 in modified Wilms-MOPS medium (20 g/L glucose) with 400 mM MOPS buffer. 8 mL filling volume in 250 mL RAMOS flasks, initial OD_600_ 0.5, 30 °C, 350 rpm at 50 mm shaking diameter. The oxygen transfer rate was monitored using a RAMOS device. Induction using 0, 0.25, or 0.5 mM IPTG at OD_600_ 1.5. **a** The oxygen transfer rate over time: the arrow indicates the induction time. For clarity, only every third data point is shown as a symbol. Shadows indicate the minimum/maximum for n = 2. **b** Total glucose consumption after 19 h, determined via linear fit in Fig. 2b. Error bars indicate minimum/maximum for n = 2 replicates. Dotted line: 20 g/L glucose was added to the medium. **c** Inulosucrase activity after 19 h (determined offline in triplicates). Statistically significant differences were determined via a two-sided t-test, * p < 0.05, ** p < 0.01, *** p < 0.005. **d** Enzyme yield was calculated using 20 g/L glucose. Error bars were calculated via Gaussian error propagation

As a basis for optimizing inulosucrase production in *V. natriegens*, the expression of the target protein InuBG-V3 was first investigated in a batch process. The effect of the inducer isopropyl-β-thiogalactoside (IPTG) at a concentration of 0.00, 0.25, and 0.50 mM on the yield of inulosucrase was tested (Fig. 5). The induction was conducted at an OD_600_ of 1.5 (indicated by a black arrow in Fig. 5a).

The OTR signals of all cultivations show three different peaks. The first OTR peak indicates the consumption of glucose. The second and third peaks indicate the consumption of overflow metabolites after glucose depletion, such as acetate, or products of mixed acid formation like lactate [4, 8, 15, 36]. No distinct differences are visible between the OTRs of the induced and not induced cultures. According to the literature [81, 82], shifts in the OTR indicating a (difference in) metabolic burden due to recombinant protein expression have been observed in *E. coli* and *Pichia pastoris*. However, no distinct differences are visible between the OTRs of the induced and non-induced cultures in Fig. 5a, even if the endpoint measurements of (inulosucrase) enzyme activity differ between the cultures (Fig. 5c) and the phenomenon of a metabolic burden has previously been reported for *V. natriegens* [83, 84]. The highest enzyme activity of 80 U/mL was obtained with the highest inducer concentration of 0.50 mM. Even higher IPTG concentrations did not positively affect *V. natriegens* when induced at an OD_600_ of 1 (Fig. S9). Becker et al. [85] also stated that no influence on protein production was found at elevated IPTG concentrations.

Remarkably, an enzyme activity of 40 U/mL was detected in the non-induced cultures. As recombinant expression of the same construct was not as leaky in *E. coli* (identical plasmid, Fig. S10), it is unlikely that the phenomenon is (only) due to the protein InuGB-V3. Tschirhart et al. [6] reported a similar observation: Their IPTG-inducible promoter was remarkably leaky in *V. natriegens* ATCC 14048, for some of the recombinant proteins expressed in the study. When the same recombinant proteins were expressed under the control of an arabinose-inducible promotor, the expression levels without induction were strongly reduced. This agrees well with the findings of Schleicher et al. [86], who did not observe leaky expression using an arabinose-inducible system with *V. natriegens* ATCC 14048. The commercial strain *V. natriegens* Vmax is derived from ATCC 14048 and contains a genomically integrated copy of the T7 polymerase under the control of the lac promotor [8]. Consequently, the strain is used for IPTG-inducible expression in many studies [11, 85, 87–91]. While the change of protein titers over the cultivation time is regularly discussed, noticeably, none of these studies presented titers of a non-induced control. At most, a negative control without plasmid is shown for comparison [88, 89].

The soft sensor (Fig. 2b) was applied to calculate the glucose consumed during the cultivations. Glucose concentrations between 14.2 and 17.2 g/L were obtained. The enzyme yield U per g of glucose was calculated using the value of 20 g/L initial glucose. Since the glucose consumption was identical in all experiments, the yield shows the same trend as the enzyme activity. With increasing IPTG concentration, the enzyme yield increases. Maximum enzyme yields of 3.9 ± 0.3 × 10^3^ U/g_glucose_ were obtained for an IPTG concentration of 0.50 mM.

**Fig. 6:**
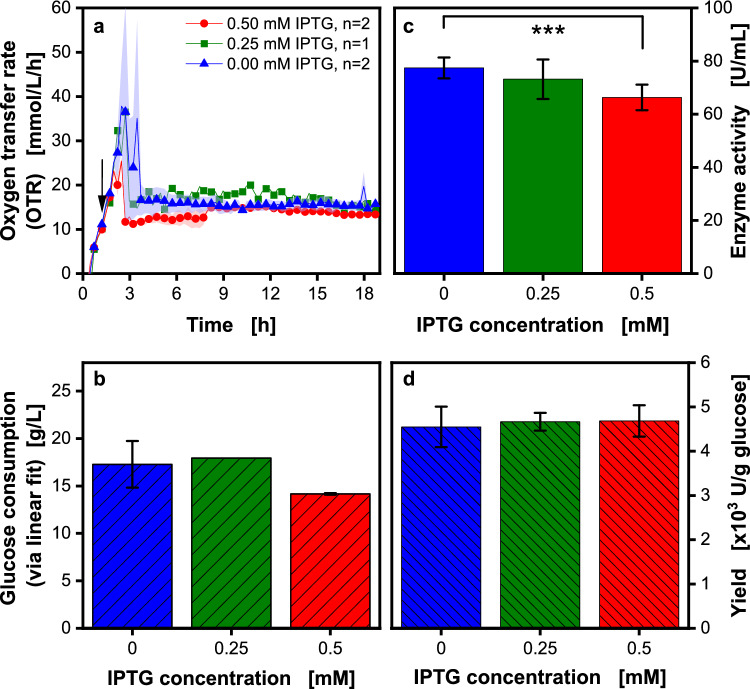
Influence of the inducer concentration (IPTG) on inulosucrase production in a fed-batch process in shake flasks*. V. natriegens* Vmax pET19b::inuGB-V3 in modified Wilms-MOPS medium (no initial glucose, 300 g/L glucose in reservoir) with 400 mM MOPS buffer. 10 mL filling volume in 250 mL RAMOS flasks, initial OD_600_ 0.5, 30 °C, 350 rpm at 50 mm shaking diameter. The oxygen transfer rate was monitored using a RAMOS device. Induction using 0, 0.25, or 0.5 mM IPTG at OD_600_ 1.5. **a** Oxygen transfer rate over time, arrow indicates the time of induction. For clarity, only every third data point is shown as a symbol. Shadows indicate the minimum/maximum for n = 2 replicates. **b** Total glucose consumption after 19 h, determined after linear fit in Fig. 2b. Error bars indicate minimum/maximum for n = 2 replicates. **c** Inulosucrase activity after 19 h (determined offline in triplicates). **d** Enzyme yield is calculated from the values in **b**, **c**. Error bars were calculated via Gaussian error propagation

As previously discussed and seen in Fig. 5, overflow metabolism and mixed acid formation are prominent in *V. natriegens* batch processes (see HPLC data in Fig. S11). For further investigation, a fed-batch in shake flasks was conducted (Fig. 6). Like before, IPTG concentrations of 0.00, 0.25, and 0.50 mM were applied for induction to compare the fed-batch to the batch (Fig. 5) process. The arrow in Fig. 6a indicates the induction time at OD_600_ 1.5.

The OTR curves of the three cultures show a batch phase in the first 3 h and a fed-batch phase in the following hours. During the batch phase, all cultures show two OTR peaks. The second peak is again due to the reuptake of acetate [4, 8, 15] that is completely consumed before the fed-batch phase. During the feeding phase, the OTR forms a plateau at approx. 15.2 ± 1.9 mmol/L/h. Just as in the batch process, no metabolic burden [66, 81, 82]is detected through the OTR signal, as previously described in the literature [66, 81, 82]. However, metabolic burden might still be present even if it does not become visible in the OTR. Overall, the membrane-based fed-batch cultivations showed good repeatability (16 replicates shown in Fig. S12). Moreover, a scale-up experiment for comparison between fed-batch cultivation in shake flask and fermenter scale showed good comparability regarding measured dissolved oxygen tension (DOT, Fig. S13). Here, the course of the DOT in the flask and in the fermenter are almost identical in the initial batch phase and very comparable within the first 12-h window.

The glucose consumption in Fig. 6b was again calculated with the soft sensor shown in Fig. 2b. During the cultivations containing 0.00 or 0.50 mM IPTG, the glucose consumption was higher than those containing 0.25 mM IPTG.

The enzyme activity in Fig. 6c shows a reverse order compared to the batch process. Cultures without IPTG resulted in the highest enzyme activity. The promoter seems even more leaky than in the batch cultivations [6]. For *E. coli*, heat shock-like stress responses to toxic IPTG concentrations have been reported by Kosinski et al. [92] and Dvorak et al. [93]. A possible hypothesis could be that *V. natriegens* behaves similarly, which could reduce the induced cultures' protein production. To gain insights into the mechanism, a future metabolomics study would be necessary.

When comparing the yields for the different IPTG concentrations (Fig. 6d**)**, the 0.25 and 0.50 mM IPTG cultures are comparable. This is because the amount of glucose consumed in the 0.25 mM culture, considered for calculating U/g_glucose_, is lower than in the other cultures. A comparable yield can be achieved by switching from an induced batch to a non-induced fed-batch process. However, lower glucose concentrations in fed-batch processes reduce the formation of overflow metabolites like acetate that have to be taken up again for full metabolization of the carbon sources. Hence, connected energy conversion losses can be circumvented. Therefore, a higher yield was originally expected in fed-batch. Inclusion bodies as a reason for the lower yields were excluded, as the sodium dodecyl sulfate—polyacrylamide gel electrophoresis (SDS-PAGE) carried out during the study showed no discrepancy with the activities determined with the Real-time GOPOD assay (Fig. S14). In addition, Kormanova et al. [11] showed that *V. natriegens* can process proteins above 60 kDA better than smaller ones. Smith et al. [94] showed significantly improved protein folding in *V. natriegens* at 30 °C. Since the enzyme yields in the fed-batch process were not as high as anticipated, a deeper understanding of the process became necessary.

**Fig. 7:**
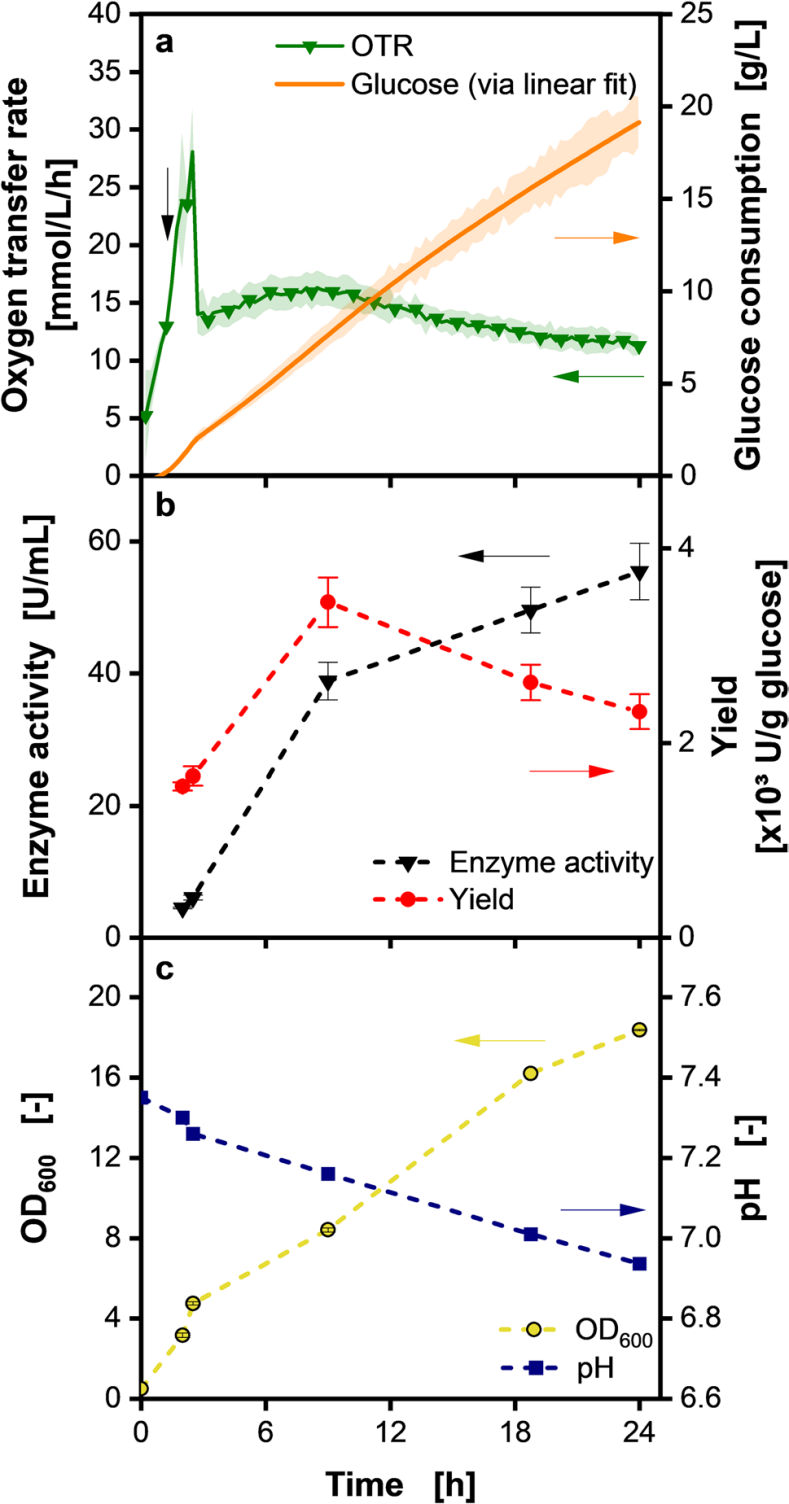
Inulosucrase expression during membrane-based fed-batch cultivation in shake flasks. Fed-batch cultivation of *V. natriegens* Vmax pET19b::inuGB-V3 in modified Wilms-MOPS medium with no initial glucose with 400 mM MOPS buffer. Induction with 0.25 mM IPTG at OD_600_ 1.5. 10 mL filling volume in 250 mL RAMOS flasks, 300 g/L glucose concentration in feed reservoir, initial OD_600_ 0.5, 37 °C, 350 rpm at 50 mm shaking diameter. The oxygen transfer rate was monitored using a RAMOS device. **a** Left axis: Oxygen transfer rate over time; a vertical arrow indicates the induction time. For clarity, only every fifth data point is shown as a symbol. Shadow indicates standard deviation for n = 3 replicates. Right axis: Glucose consumption determined via the linear fit presented in Fig. 2b (orange line). **b** Left axis: Enzymatic activity of the product inulosucrase measured offline. Error bars indicate standard deviation for n = 3 replicates. Right axis: Substrate yield per g of consumed glucose determined via linear fit. **c** Left axis: OD_600_. Right axis: pH offline measured (blue symbols)

We analyzed samples collected throughout the fed-batch fermentation time to investigate whether *V. natriegens* degrades inulosucrase over time (Fig. 7). The online measurement of the OTR (Fig. 7a) indicates a batch and a fed-batch phase. The arrow marks the induction time at OD_600_ 1.5 with an inducer concentration of 0.25 mM IPTG. The production rate appears to decrease when looking at the enzyme activities in the offline samples after 8.5 h (see Fig. 7b).

During the fed-batch fermentation shown in Fig. 7c, the glucose consumption rate is linear. As more biomass accumulates over time, more glucose is required for maintenance metabolism. Since the enzyme activity increases during cultivation (Fig. 7b), it is unlikely that it is degraded by *Vibrio natriegens*. However, the enzyme production rate declines over time, resulting in a declining yield. Due to the decrease in production rate, longer fed-batch fermentations were not anticipated to result in higher product yields.

The OD_600_ (Fig. 7c) increases during the first 2.5 h to an OD_600_ of 4.7. Afterwards, the OD_600_ increases linearly during the fed-batch and reaches a final OD_600_ of 18.4 after 24 h. This pattern is due to the linear feed rate and known from previous fed-batch processes in shake flasks [45, 64]. On initial observation, the final OD_600_ of 18.4 appears to be relatively low. However, upon closer comparison of the batch (Fig. S11) and fed-batch data, it becomes evident that while comparable final OD_600_ values are obtained in the batch process, the enzyme activities are substantially lower. Consequently, a greater proportion of substrate is converted into product in the fed-batch process than in the batch process. Exopolysaccharide formation as reported by Schulze et al. [73] was observed in this study in samples at later time points (Fig. S15). However, no interference with the process was noted, and the exopolysaccharide formation was not investigated further.

The pH decreases during the batch phase from a pH_start_ of 7.35 to a pH of 7.26 after 2.5 h due to acidic metabolite formation. During the fed-batch phase, the pH decreases linearly to a pH of 7.01 after 18 h and to a final value of 6.94 after 24 h due to ammonia uptake [15]. The pH after 18 h matches the value of 7.01 shown in Fig. 4c (300 g/L reservoir concentration, sampling after 18 h) perfectly. Over the whole process, the pH stays in the preferred range of *V. natriegens* [2, 95].

Although the yield of the fed-batch fermentations (Fig. 6d) does not exceed that of the batch fermentations (Fig. 5d), fed-batch fermentations with shake flasks are still advantageous: Feeding a limited amount of glucose leads to reduced overflow metabolism during cultivation, thereby resulting in a stabilized pH. In this study, a model strain was used for fed-batch process development. In the future, this protocol may be a useful tool for the screening of *V. natriegens* strains to identify the most suitable candidate for an industrial process.

## Conclusion

This study successfully outlined the effectiveness of small-scale fed-batch cultivations of *V. natriegens* using 96-well FeedPlates® and membrane-based fed-batch shake flasks. Excellent repeatability of the membrane-based fed-batch technology in combination with *V. natriegens* was demonstrated. Switching from batch to fed-batch processes significantly minimized overflow metabolism and prevented mixed acid formation for linear glucose feed rates between 1.1 and 1.8 g/L/h (Fig. 4). The introduction of a glucose soft sensor, based on the online-measured oxygen transfer rate (OTR) detected with the µTOM device, provided a reliable and noninvasive means to estimate glucose consumption and, consequently, feed rates in fed-batch processes.

After establishing small-scale fed-batch cultivations, the recombinant production of the inulosucrase InuBG-V3 was investigated. Varying the inducer IPTG showed significant concentration-dependent effects in batch processes, a phenomenon not observed in fed-batch processes. Expression controlled by the lac promoter was remarkably leaky in fed-batch processes, resulting in high inulosucrase activity. Overall, the expression of InuGB-V3 in *V. natriegens* resulted in an inulosucrase titer of 80 U/mL.

Our results confirm that *V. natriegens* can serve as a biotechnological workhorse with high potential as an economical alternative to *E. coli*. Further in-depth studies are required to fully elucidate and optimize the presented cultivation technologies for broader applications. For example, the dependency of expression on induction conditions in batch and fed-batch should be further investigated by induction profiling [82, 96]. In addition, alternative induction strategies could be relevant where the IPTG concentration is continuously or stepwise increased. A better understanding of the metabolism of *V. natriegens* at low glucose availability is essential. In particular, testing wild types against prophage-free strains should prove informative.

## Supplementary Information

Below is the link to the electronic supplementary material.

Supplementary file1 (PDF 1615 KB)

## Data Availability

All data is included within the manuscript and its supplementary file. Data in a machine-readable format is available from the corresponding author upon reasonable request.

## References

[1] Xu J, Dong F, Wu M et al (2021) *Vibrio**natriegens* as a pET-compatible expression host complementary to *Escherichia coli*. Front Microbiol 12:627181. 10.3389/fmicb.2021.627181 33679648 10.3389/fmicb.2021.627181 PMC7933001

[2] Hoff J, Daniel B, Stukenberg D et al (2020) *Vibrio**natriegens*: an ultrafast-growing marine bacterium as emerging synthetic biology chassis. Environ Microbiol 22:4394–4408. 10.1111/1462-2920.15128 32537803 10.1111/1462-2920.15128

[3] Ellis GA, Tschirhart T, Spangler J et al (2019) Exploiting the feedstock flexibility of the emergent synthetic biology chassis *Vibrio**natriegens* for engineered natural product production. Mar Drugs. 10.3390/md17120679 31801279 10.3390/md17120679 PMC6950413

[4] Hoffart E, Grenz S, Lange J et al (2017) High substrate uptake rates empower *Vibrio natriegens* as production host for industrial biotechnology. Appl Environ Microbiol. 10.1128/AEM.01614-17 28887417 10.1128/AEM.01614-17 PMC5666143

[5] Eagon RG (1961) *Pseudomonas**natriegens*, a marine bacterium with a generation time of less than 10 minutes. J Bacteriol. 10.1128/jb.83.4.736-737.1962 13888946 10.1128/jb.83.4.736-737.1962 PMC279347

[6] Tschirhart T, Shukla V, Kelly EE et al (2019) Synthetic biology tools for the fast-growing marine bacterium *Vibrio**natriegens*. ACS Synth Biol 8:2069–2079. 10.1021/acssynbio.9b00176 31419124 10.1021/acssynbio.9b00176

[7] Thoma F, Schulze C, Gutierrez-Coto C et al (2022) Metabolic engineering of *Vibrio**natriegens* for anaerobic succinate production. Microb Biotechnol 15:1671–1684. 10.1111/1751-7915.13983 34843164 10.1111/1751-7915.13983 PMC9151343

[8] Thoma F, Blombach B (2021) Metabolic engineering of *Vibrio**natriegens*. Essays Biochem 65:381–392. 10.1042/EBC20200135 33835156 10.1042/EBC20200135 PMC8314017

[9] Weinstock MT, Hesek ED, Wilson CM et al (2016) *Vibrio**natriegens* as a fast-growing host for molecular biology. Nat Methods 13:849–851. 10.1038/nmeth.3970 27571549 10.1038/nmeth.3970

[10] Coppens L, Tschirhart T, Leary DH et al (2023) *Vibrio**natriegens* genome-scale modeling reveals insights into halophilic adaptations and resource allocation. Mol Syst Biol 19:e10523. 10.15252/msb.202110523 36847213 10.15252/msb.202110523 PMC10090949

[11] Kormanová Ľ, Rybecká S, Levarski Z et al (2020) Comparison of simple expression procedures in novel expression host *Vibrio**natriegens* and established *Escherichia**coli* system. J Biotechnol 321:57–67. 10.1016/j.jbiotec.2020.06.003 32589894 10.1016/j.jbiotec.2020.06.003

[12] Wiegand DJ, Lee HH, Ostrov N et al (2018) Establishing a cell-free *Vibrio**natriegens* expression system. ACS Synth Biol 7:2475–2479. 10.1021/acssynbio.8b00222 30160938 10.1021/acssynbio.8b00222

[13] Fernández-Llamosas H, Castro L, Blázquez ML et al (2017) Speeding up bioproduction of selenium nanoparticles by using *Vibrio**natriegens* as microbial factory. Sci Rep 7:16046. 10.1038/s41598-017-16252-1 29167550 10.1038/s41598-017-16252-1 PMC5700131

[14] Meng W, Zhang Y, Ma L et al (2022) Non-sterilized fermentation of 2,3-butanediol with seawater by metabolic engineered fast-growing *Vibrio**natriegens*. Front Bioeng Biotechnol 10:955097. 10.3389/fbioe.2022.955097 35903792 10.3389/fbioe.2022.955097 PMC9315368

[15] Thiele I, Gutschmann B, Aulich L et al (2021) High-cell-density fed-batch cultivations of *Vibrio**natriegens*. Biotechnol Lett 43:1723–1733. 10.1007/s10529-021-03147-5 34009528 10.1007/s10529-021-03147-5 PMC8397650

[16] Stella RG, Baumann P, Lorke S et al (2021) Biosensor-based isolation of amino acid-producing *Vibrio**natriegens* strains. Metab Eng Commun 13:e00187. 10.1016/j.mec.2021.e00187 34824977 10.1016/j.mec.2021.e00187 PMC8605253

[17] Peter CP, Suzuki Y, Rachinskiy K et al (2006) Volumetric power consumption in baffled shake flasks. Chem Eng Sci 61:3771–3779. 10.1016/j.ces.2005.12.020

[18] Peter CP, Suzuki Y, Büchs J (2006) Hydromechanical stress in shake flasks: correlation for the maximum local energy dissipation rate. Biotechnol Bioeng 93:1164–1176. 10.1002/bit.20827 16470882 10.1002/bit.20827

[19] Büchs J (2001) Introduction to advantages and problems of shaken cultures. Biochem Eng J 7:91–98. 10.1016/s1369-703x(00)00106-6 11173295 10.1016/s1369-703x(00)00106-6

[20] Tan R-K, Eberhard W, Büchs J (2011) Measurement and characterization of mixing time in shake flasks. Chem Eng Sci 66:440–447. 10.1016/j.ces.2010.11.001

[21] Palacios-Morales C, Aguayo-Vallejo JP, Trujillo-Roldán MA et al (2016) The flow inside shaking flasks and its implication for mycelial cultures. Chem Eng Sci 152:163–171. 10.1016/j.ces.2016.06.016

[22] Peter CP, Lotter S, Maier U et al (2004) Impact of out-of-phase conditions on screening results in shaking flask experiments. Biochem Eng J 17:205–215. 10.1016/S1369-703X(03)00179-7

[23] Büchs J, Maier U, Lotter S et al (2007) Calculating liquid distribution in shake flasks on rotary shakers at waterlike viscosities. Biochem Eng J 34:200–208. 10.1016/j.bej.2006.12.005

[24] van Suijdam JC, Kossen NWF, Joha AC (1978) Model for oxygen transfer in a shake flask. Biotechnol Bioeng 20:1695–1709. 10.1002/bit.260201102

[25] Büchs J, Lotter S, Milbradt C (2001) Out-of-phase operating conditions, a hitherto unknown phenomenon in shaking bioreactors. Biochem Eng J 7:135–141. 10.1016/s1369-703x(00)00113-3 11173302 10.1016/s1369-703x(00)00113-3

[26] Maier U, Losen M, Büchs J (2004) Advances in understanding and modeling the gas–liquid mass transfer in shake flasks. Biochem Eng J 17:155–167. 10.1016/S1369-703X(03)00174-8

[27] Giese H, Azizan A, Kümmel A et al (2014) Liquid films on shake flask walls explain increasing maximum oxygen transfer capacities with elevating viscosity. Biotechnol Bioeng 111:295–308. 10.1002/bit.25015 23904288 10.1002/bit.25015

[28] Hermann R, Lehmann M, Büchs J (2003) Characterization of gas-liquid mass transfer phenomena in microtiter plates. Biotechnol Bioeng 81:178–186. 10.1002/bit.10456 12451554 10.1002/bit.10456

[29] Nikakhtari H, Hill GA (2005) Modelling oxygen transfer and aerobic growth in shake flasks and well-mixed bioreactors. Can J Chem Eng 83:493–499. 10.1002/cjce.5450830312

[30] Meier K, Klöckner W, Bonhage B et al (2016) Correlation for the maximum oxygen transfer capacity in shake flasks for a wide range of operating conditions and for different culture media. Biochem Eng J 109:228–235. 10.1016/j.bej.2016.01.014

[31] Funke M, Buchenauer A, Mokwa W et al (2010) Bioprocess control in microscale: scalable fermentations in disposable and user-friendly microfluidic systems. Microb Cell Fact 9:86. 10.1186/1475-2859-9-86 21073740 10.1186/1475-2859-9-86 PMC3000389

[32] Kensy F, Engelbrecht C, Büchs J (2009) Scale-up from microtiter plate to laboratory fermenter: evaluation by online monitoring techniques of growth and protein expression in *Escherichia**coli* and *Hansenula**polymorpha* fermentations. Microb Cell Fact 8:68. 10.1186/1475-2859-8-68 20028556 10.1186/1475-2859-8-68 PMC2806293

[33] Neuss A, Steimann T, Tomas Borges JS et al (2025) Scale-up of CHO cell cultures: from 96-well-microtiter plates to stirred tank reactors across three orders of magnitude. J Biol Eng 19:5. 10.1186/s13036-024-00475-8 39815355 10.1186/s13036-024-00475-8 PMC11734472

[34] Seletzky JM, Noack U, Hahn S et al (2007) An experimental comparison of respiration measuring techniques in fermenters and shake flasks: exhaust gas analyzer vs. RAMOS device vs. respirometer. J Ind Microbiol Biotechnol 34:123–130. 10.1007/s10295-006-0176-2 17001475 10.1007/s10295-006-0176-2

[35] Dinger R, Lattermann C, Flitsch D et al (2022) Device for respiration activity measurement enables the determination of oxygen transfer rates of microbial cultures in shaken 96-deepwell microtiter plates. Biotechnol Bioeng 119:881–894. 10.1002/bit.28022 34951007 10.1002/bit.28022

[36] Anderlei T, Büchs J (2001) Device for sterile online measurement of the oxygen transfer rate in shaking flasks. Biocheml Eng J. 10.1016/s1369-703x(00)00116-9 10.1016/s1369-703x(00)00116-9 11173305

[37] Garcia-Ochoa F, Gomez E, Santos VE et al (2010) Oxygen uptake rate in microbial processes: an overview. Biochem Eng J 49:289–307. 10.1016/j.bej.2010.01.011

[38] Anderlei T, Zang W, Papaspyrou M et al (2004) Online respiration activity measurement (OTR, CTR, RQ) in shake flasks. Biochem Eng J 17:187–194. 10.1016/S1369-703X(03)00181-5

[39] Losen M, Frölich B, Pohl M et al (2004) Effect of oxygen limitation and medium composition on *Escherichia**coli* fermentation in shake-flask cultures. Biotechnol Prog 20:1062–1068. 10.1021/bp034282t 15296430 10.1021/bp034282t

[40] Wewetzer SJ, Kunze M, Ladner T et al (2015) Parallel use of shake flask and microtiter plate online measuring devices (RAMOS and BioLector) reduces the number of experiments in laboratory-scale stirred tank bioreactors. J Biol Eng 9:9. 10.1186/s13036-015-0005-0 26265936 10.1186/s13036-015-0005-0 PMC4531433

[41] Buchenauer A, Hofmann MC, Funke M et al (2009) Micro-bioreactors for fed-batch fermentations with integrated online monitoring and microfluidic devices. Biosens Bioelectron 24:1411–1416. 10.1016/j.bios.2008.08.043 18929478 10.1016/j.bios.2008.08.043

[42] Habicher T, Czotscher V, Klein T et al (2019) Glucose-containing polymer rings enable fed-batch operation in microtiter plates with parallel online measurement of scattered light, fluorescence, dissolved oxygen tension, and pH. Biotechnol Bioeng 116:2250–2262. 10.1002/bit.27077 31161630 10.1002/bit.27077

[43] Toeroek C, Cserjan-Puschmann M, Bayer K et al (2015) Fed-batch like cultivation in a micro-bioreactor: screening conditions relevant for *Escherichia**coli* based production processes. Springerplus 4:490. 10.1186/s40064-015-1313-z 26380166 10.1186/s40064-015-1313-z PMC4567571

[44] Biener R, Horn T, Komitakis A et al (2023) High-cell-density cultivation of *Vibrio**natriegens* in a low-chloride chemically defined medium. Appl Microbiol Biotechnol 107:7043–7054. 10.1007/s00253-023-12799-4 37741940 10.1007/s00253-023-12799-4 PMC10638117

[45] Jeude M, Dittrich B, Niederschulte H et al (2006) Fed-batch mode in shake flasks by slow-release technique. Biotechnol Bioeng 95:433–445. 10.1002/bit.21012 16736531 10.1002/bit.21012

[46] Keil T, Landenberger M, Dittrich B et al (2019) Precultures grown under fed-batch conditions increase the reliability and reproducibility of high-throughput screening results. Biotechnol J 14:e1800727. 10.1002/biot.201800727 31283111 10.1002/biot.201800727

[47] Panula-Perälä J, Siurkus J, Vasala A et al (2008) Enzyme controlled glucose auto-delivery for high cell density cultivations in microplates and shake flasks. Microb Cell Fact 7:31. 10.1186/1475-2859-7-31 19017379 10.1186/1475-2859-7-31 PMC2588551

[48] Cui N, Pozzobon V (2022) Food-grade cultivation of *Saccharomyces cerevisiae* from potato waste. AgriEngineering 4:951–968. 10.3390/agriengineering4040061

[49] Keil T, Dittrich B, Rührer J et al (2019) Polymer-based ammonium-limited fed-batch cultivation in shake flasks improves lipid productivity of the microalga *Chlorella**vulgaris*. Bioresour Technol 291:121821. 10.1016/j.biortech.2019.121821 31352167 10.1016/j.biortech.2019.121821

[50] Bähr C, Leuchtle B, Lehmann C et al (2012) Dialysis shake flask for effective screening in fed-batch mode. Biochem Eng J 69:182–195. 10.1016/j.bej.2012.08.012

[51] Philip P, Kern D, Goldmanns J et al (2018) Parallel substrate supply and pH stabilization for optimal screening of *E.**coli* with the membrane-based fed-batch shake flask. Microb Cell Fact 17:69. 10.1186/s12934-018-0917-8 29743073 10.1186/s12934-018-0917-8 PMC5941677

[52] Wu F, Wang S, Peng Y et al (2023) Metabolic engineering of fast-growing *Vibrio natriegens* for efficient pyruvate production. Microb Cell Fact 22:172. 10.1186/s12934-023-02185-0 37667234 10.1186/s12934-023-02185-0 PMC10476420

[53] Zhang Y, Li Z, Liu Y et al (2021) Systems metabolic engineering of *Vibrio natriegens* for the production of 1,3-propanediol. Metab Eng 65:52–65. 10.1016/j.ymben.2021.03.008 33722653 10.1016/j.ymben.2021.03.008

[54] Keil T, Dittrich B, Lattermann C et al (2020) Optimized polymer-based glucose release in microtiter plates for small-scale *E.**coli* fed-batch cultivations. J Biol Eng 14:24. 10.1186/s13036-020-00247-0 32874201 10.1186/s13036-020-00247-0 PMC7457294

[55] Müller J, Hütterott A, Habicher T et al (2019) Validation of the transferability of membrane-based fed-batch shake flask cultivations to stirred-tank reactor using three different protease producing Bacillus strains. J Biosci Bioeng 128:599–605. 10.1016/j.jbiosc.2019.05.003 31151898 10.1016/j.jbiosc.2019.05.003

[56] Pohlentz JC, Gallala N, Kosciow K et al (2022) Growth behavior of probiotic microorganisms on levan- and inulin-based fructans. J Funct Foods 99:105343. 10.1016/j.jff.2022.105343

[57] Wienberg F, Hövels M, Deppenmeier U (2022) High-yield production and purification of prebiotic inulin-type fructooligosaccharides. AMB Express 12:144. 10.1186/s13568-022-01485-9 36380213 10.1186/s13568-022-01485-9 PMC9666576

[58] Sánchez-Martínez MJ, Soto-Jover S, Antolinos V et al (2020) Manufacturing of short-chain fructooligosaccharides: from laboratory to industrial scale. Food Eng Rev 12:149–172. 10.1007/s12393-020-09209-0

[59] Nobre C, Simões LS, Gonçalves DA et al (2022) Fructooligosaccharides production and the health benefits of prebiotics. Current developments in biotechnology and bioengineering. Elsevier, Amsterdam, pp 109–138

[60] Wienberg F, Hövels M, Kosciow K et al (2021) High-resolution method for isocratic HPLC analysis of inulin-type fructooligosaccharides. J Chromatogr B Analyt Technol Biomed Life Sci 1172:122505. 10.1016/j.jchromb.2020.122505 33895646 10.1016/j.jchromb.2020.122505

[61] Keil T, Dittrich B, Lattermann C et al (2019) Polymer-based controlled-release fed-batch microtiter plate—diminishing the gap between early process development and production conditions. J Biol Eng 13:18. 10.1186/s13036-019-0147-6 30833982 10.1186/s13036-019-0147-6 PMC6387502

[62] Habicher T, Klein T, Becker J et al (2021) Screening for optimal protease producing *Bacillus**licheniformis* strains with polymer-based controlled-release fed-batch microtiter plates. Microb Cell Fact 20:51. 10.1186/s12934-021-01541-2 33622330 10.1186/s12934-021-01541-2 PMC7903736

[63] Habicher T, John A, Scholl N et al (2019) Introducing substrate limitations to overcome catabolite repression in a protease producing *Bacillus**licheniformis* strain using membrane-based fed-batch shake flasks. Biotechnol Bioeng 116:1326–1340. 10.1002/bit.26948 30712275 10.1002/bit.26948

[64] Philip P, Meier K, Kern D et al (2017) Systematic evaluation of characteristics of the membrane-based fed-batch shake flask. Microb Cell Fact 16:122. 10.1186/s12934-017-0741-6 28716035 10.1186/s12934-017-0741-6 PMC5514527

[65] Wilms B, Hauck A, Reuss M et al (2001) High-cell-density fermentation for production of L-N-carbamoylase using an expression system based on the *Escherichia**coli* rhaBAD promoter. Biotechnol Bioeng 73:95–103. 10.1002/bit.1041 11255157 10.1002/bit.1041

[66] Mühlmann MJ, Forsten E, Noack S et al (2018) Prediction of recombinant protein production by *Escherichia**coli* derived online from indicators of metabolic burden. Biotechnol Prog 34:1543–1552. 10.1002/btpr.2704 30248250 10.1002/btpr.2704

[67] Forsten E, Gerdes S, Petri R et al (2024) Unraveling the impact of pH, sodium concentration, and medium osmolality on Vibrio natriegens in batch processes. BMC Biotechnol 24:63. 10.1186/s12896-024-00897-8 39313794 10.1186/s12896-024-00897-8 PMC11421182

[68] Kensy F, Zang E, Faulhammer C et al (2009) Validation of a high-throughput fermentation system based on online monitoring of biomass and fluorescence in continuously shaken microtiter plates. Microb Cell Fact 8:31. 10.1186/1475-2859-8-31 19497126 10.1186/1475-2859-8-31 PMC2700080

[69] Azoddein AAM, Ahmad MM, Yunus RM et al (2017) Effect of acclimatization time to microbial cell growth and biosynthesis of mesophilic gammaproteobacterium, in orbital shake flasks. MATEC Web Conf 109:4003. 10.1051/matecconf/201710904003

[70] Ehinger FJ, Neff A, Kosciow K et al (2022) Rapid, real-time sucrase characterization: showcasing the feasibility of a one-pot activity assay. J Biotechnol 354:21–33. 10.1016/j.jbiotec.2022.06.004 35716887 10.1016/j.jbiotec.2022.06.004

[71] Erian AM, Freitag P, Gibisch M et al (2020) High rate 2,3-butanediol production with *Vibrio**natriegens*. Bioresour Technol Rep 10:100408. 10.1016/j.biteb.2020.100408

[72] Long CP, Gonzalez JE, Cipolla RM et al (2017) Metabolism of the fast-growing bacterium *Vibrio**natriegens* elucidated by 13C metabolic flux analysis. Metab Eng 44:191–197. 10.1016/j.ymben.2017.10.008 29042298 10.1016/j.ymben.2017.10.008 PMC5845447

[73] Schulze C, Hädrich M, Borger J et al (2023) Investigation of exopolysaccharide formation and its impact on anaerobic succinate production with *Vibrio natriegens*. Microb Biotechnol. 10.1111/1751-7915.14277 37256270 10.1111/1751-7915.14277 PMC10832516

[74] Duetz WA, Rüedi L, Hermann R et al (2000) Methods for intense aeration, growth, storage, and replication of bacterial strains in microtiter plates. Appl Environ Microbiol 66:2641–2646. 10.1128/AEM.66.6.2641-2646.2000 10831450 10.1128/aem.66.6.2641-2646.2000 PMC110593

[75] Ljunggren J, Häggström L (1992) Glutamine limited fed-batch culture reduces the overflow metabolism of amino acids in myeloma cells. Cytotechnology. 10.1007/bf02540029 1368403 10.1007/BF02540029

[76] Dragosits M, Frascotti G, Bernard-Granger L et al (2011) Influence of growth temperature on the production of antibody Fab fragments in different microbes: a host comparative analysis. Biotechnol Prog 27:38–46. 10.1002/btpr.524 21312353 10.1002/btpr.524

[77] de Groot NS, Ventura S (2006) Effect of temperature on protein quality in bacterial inclusion bodies. FEBS Lett 580:6471–6476. 10.1016/j.febslet.2006.10.071 17101131 10.1016/j.febslet.2006.10.071

[78] Rosano GL, Ceccarelli EA (2014) Recombinant protein expression in *Escherichia**coli*: advances and challenges. Front Microbiol 5:172. 10.3389/fmicb.2014.00172 24860555 10.3389/fmicb.2014.00172 PMC4029002

[79] Mogi N, Sugai E, Fuse Y et al (2007) Infinite dilution binary diffusion coefficients for six sugars at 0.1 MPa and temperatures from (273.2 to 353.2) K. J Chem Eng Data 52:40–43. 10.1021/je0601816

[80] Gladden JK, Dole M (1953) Diffusion in supersaturated solutions. II. Glucose solutions. J Am Chem Soc 75:3900–3904. 10.1021/ja01112a008

[81] Wollborn D, Munkler LP, Horstmann R et al (2022) Predicting high recombinant protein producer strains of *Pichia**pastoris* MutS using the oxygen transfer rate as an indicator of metabolic burden. Sci Rep 12:11225. 10.1038/s41598-022-15086-w 35780248 10.1038/s41598-022-15086-w PMC9250517

[82] Mühlmann M, Forsten E, Noack S et al (2017) Optimizing recombinant protein expression via automated induction profiling in microtiter plates at different temperatures. Microb Cell Fact 16:220. 10.1186/s12934-017-0832-4 29183374 10.1186/s12934-017-0832-4 PMC5706349

[83] Tietze L, Mangold A, Hoff MW et al (2022) Identification and cross-characterisation of artificial promoters and 5’ untranslated regions in vibrio natriegens. Front Bioeng Biotechnol 10:826142. 10.3389/fbioe.2022.826142 35155395 10.3389/fbioe.2022.826142 PMC8830501

[84] Stukenberg D, Faber A, Becker A (2024) Graded-CRISPRi, a tool for tuning the strengths of CRISPRi-mediated knockdowns in vibrio natriegens using gRNA libraries. ACS Synth Biol 13:2091–2104. 10.1021/acssynbio.4c00056 38916455 10.1021/acssynbio.4c00056

[85] Becker W, Wimberger F, Zangger K (2019) *Vibrio**natriegens*: an alternative expression system for the high-yield production of isotopically labeled proteins. Biochemistry 58:2799–2803. 10.1021/acs.biochem.9b00403 31199119 10.1021/acs.biochem.9b00403

[86] Schleicher L, Muras V, Claussen B et al (2018) Vibrio natriegens as host for expression of multisubunit membrane protein complexes. Front Microbiol 9:2537. 10.3389/fmicb.2018.02537 30410475 10.3389/fmicb.2018.02537 PMC6209661

[87] Stadler KA, Becker W, Darnhofer B et al (2022) Overexpression of recombinant proteins containing non-canonical amino acids in Vibrio natriegens: p-azido-L-phenylalanine as coupling site for 19F-tags. Amino Acids 54:1041–1053. 10.1007/s00726-022-03148-2 35419750 10.1007/s00726-022-03148-2 PMC9217835

[88] Kormanová Ľ, Levarski Z, Minich A et al (2023) Novel expression system based on enhanced permeability of Vibrio natriegens cells induced by D, D- carboxypeptidase overexpression. World J Microbiol Biotechnol 39:277. 10.1007/s11274-023-03723-z 37568013 10.1007/s11274-023-03723-z PMC10421817

[89] Fuchs H, Ullrich SR, Hedrich S (2024) Vibrio natriegens as a superior host for the production of c-type cytochromes and difficult-to-express redox proteins. Sci Rep 14:6093. 10.1038/s41598-024-54097-7 38480761 10.1038/s41598-024-54097-7 PMC10937671

[90] Mojica N, Kersten F, Montserrat-Canals M et al (2024) Using vibrio natriegens for high-yield production of challenging expression targets and for protein perdeuteration. Biochemistry 63:587–598. 10.1021/acs.biochem.3c00612 38359344 10.1021/acs.biochem.3c00612 PMC10919088

[91] Liu X, Han X, Peng Y et al (2022) Rapid production of l-DOPA by Vibrio natriegens, an emerging next-generation whole-cell catalysis chassis. Microb Biotechnol 15:1610–1621. 10.1111/1751-7915.14001 35006649 10.1111/1751-7915.14001 PMC9049612

[92] Kosinski M, Rinas U, Bailey J (1992) Isopropyl-ß-d-thiogalactopyranoside influences the metabolism of *Escherichia coli*. Appl Microbiol Biotechnol 36:782–784. 10.1007/BF00172194

[93] Dvorak P, Chrast L, Nikel PI et al (2015) Exacerbation of substrate toxicity by IPTG in *Escherichia coli BL21*(DE3) carrying a synthetic metabolic pathway. Microb Cell Fact 14:201. 10.1186/s12934-015-0393-3 26691337 10.1186/s12934-015-0393-3 PMC4687329

[94] Smith M, Hernández JS, Messing S et al (2024) Producing recombinant proteins in Vibrio natriegens. Microb Cell Fact 23:208. 10.1186/s12934-024-02455-5 39049057 10.1186/s12934-024-02455-5 PMC11267860

[95] Payne WJ, Eagon RG, Williams AK (1961) Some observations on the physiology of *Pseudomonas**natriegens* nov. spec. Antonie Van Leeuwenhoek 27:121–128. 10.1007/BF02538432 13733692 10.1007/BF02538432

[96] Wandrey G, Bier C, Binder D et al (2016) Light-induced gene expression with photocaged IPTG for induction profiling in a high-throughput screening system. Microb Cell Fact 15:63. 10.1186/s12934-016-0461-3 27107964 10.1186/s12934-016-0461-3 PMC4842301

