# Supplementary material for: Small-scale fed-batch cultivations of Vibrio natriegens: overcoming challenges for early process development
Source: Bioprocess Biosyst Eng. 2025 Apr 18;48(6):1007–24. doi: 10.1007/s00449-025-03159-9 (PMC12089209; doi:10.1007/s00449-025-03159-9)
Supplement: Supplementary file 1 — Supplementary file1 (PDF 1615 KB) [file 449_2025_3159_MOESM1_ESM.pdf]

Supplement

Small-Scale Fed-Batch Cultivations of *Vibrio natriegens*:

Overcoming Challenges for Early Process Development

Clara Lüchtrath[1,*], Eva Forsten[1,*], Romeos Polis[1], Maximilian Hoffmann[1], Aylin Sara Genis[2], Anna-Lena Kuhn[1], Marcel Hövels[2], Uwe Deppenmeier[2], Jørgen Magnus[1], Jochen Büchs[1,§]

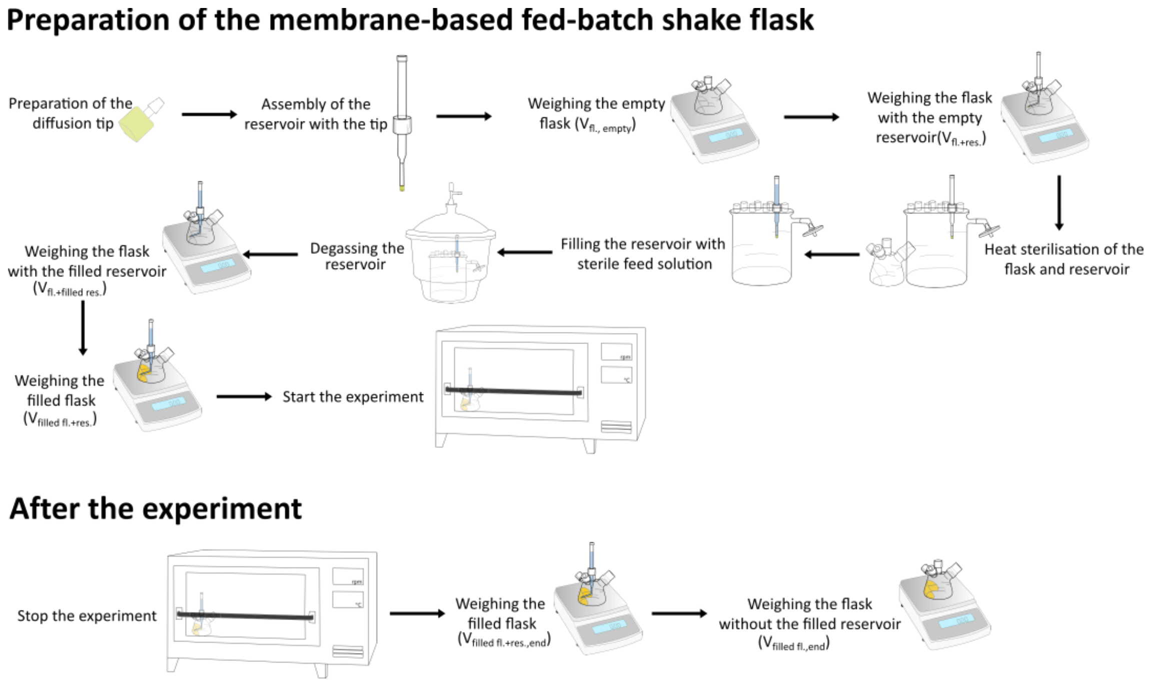

Fig. S1: Preparation of the membrane-based fed-batch shake flask.

The diluting effect of reverse water diffusion (weight difference ΔV_{res}) is calculated via the following equations.

$$V_{res,empty} = V_{fl.+res} - V_{fl.,empty}$$

$$V_{res,filled} = V_{filled\ fl.+res} - V_{fl.+res}$$

$$V_{res,end} = V_{filled\ fl.+res,end} - V_{filled\ fl.,end}$$

$$\Delta V_{res} = V_{res,end} - V_{res,filled}$$

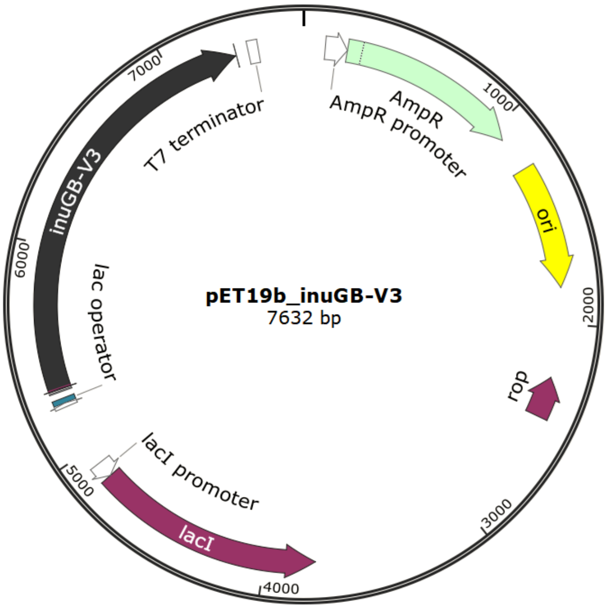

Fig. S2 Plasmid map of pET19b::inuGB-V3

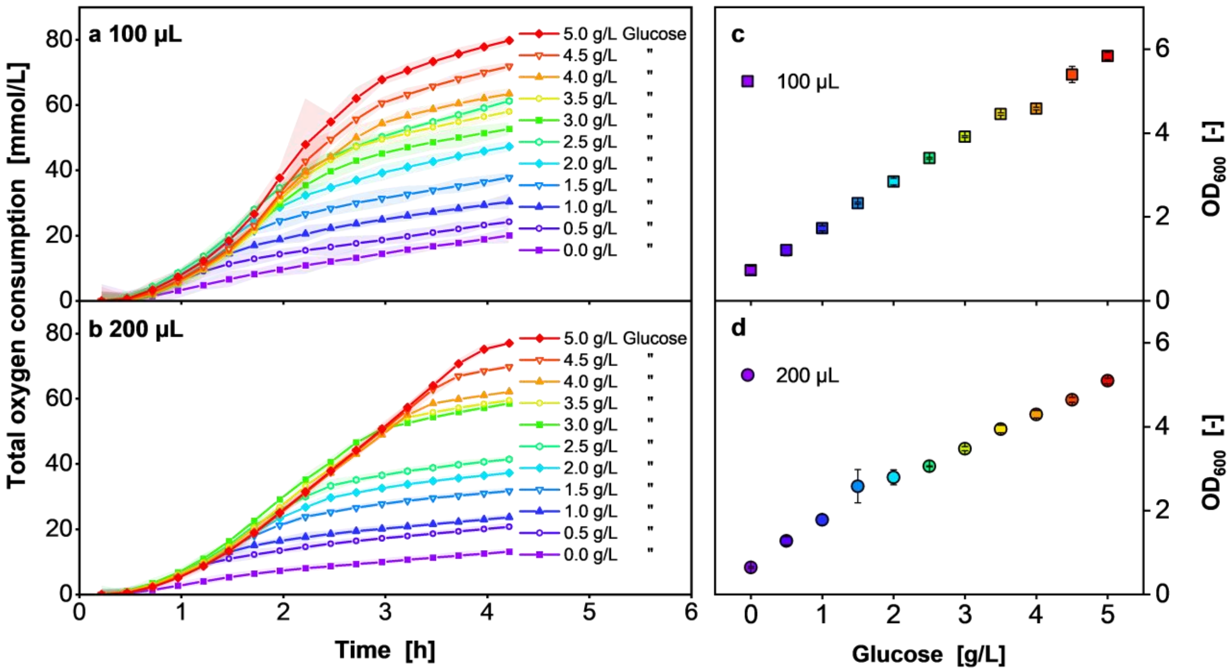

Fig. S3 Total oxygen consumption over time in a batch cultivation *V. natriegens* Vmax pET19b::inuGB-V3 in modified Wilms-MOPS medium (0-5 g/L initial glucose) with 400 mM MOPS buffer. Initial OD_{600} 0.5, 37 °C, 1000 rpm at 3 mm shaking diameter. The oxygen transfer rate was monitored using a µTOM device. (a) 100 µL filling volume in 96-deep well plate. (b) 200 µL filling volume in 96-deep well plate. The total oxygen consumption (a) and (b) are calculated via integration of Fig 2 a and Fig S4 a. (c) and (d) Offline-measured final OD_{600} depending on the glucose concentration.

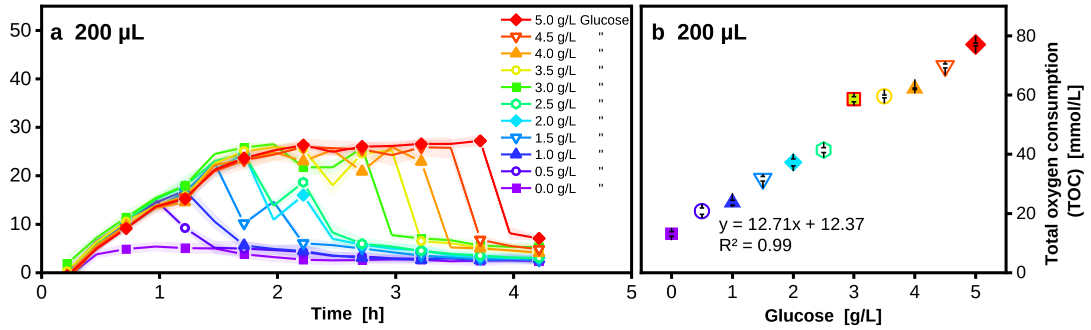

Fig. S4 Calibrating the oxygen transfer to glucose consumption in a 96-deep well plate in a batch cultivation. Non induced batch cultivation of *V. natriegens* Vmax pET19b::inuGB-V3 in modified Wilms-MOPS medium (0-5 g/L glucose) with 400 mM MOPS buffer. Initial OD*600* 0.5, 37 °C, 1000 rpm at 3 mm shaking diameter, oxygen transfer rate monitored using a µTOM device. (a) OTR of a cultivation with 200 µL filling volume in a 96-deep well plate. For clarity, only every third data point is shown as a symbol. Shadows indicate standard deviation for n = 4 replicates. (b) Linear correlation of the total oxygen consumption (shown in Fig. S3 b) to the provided and total consumed glucose

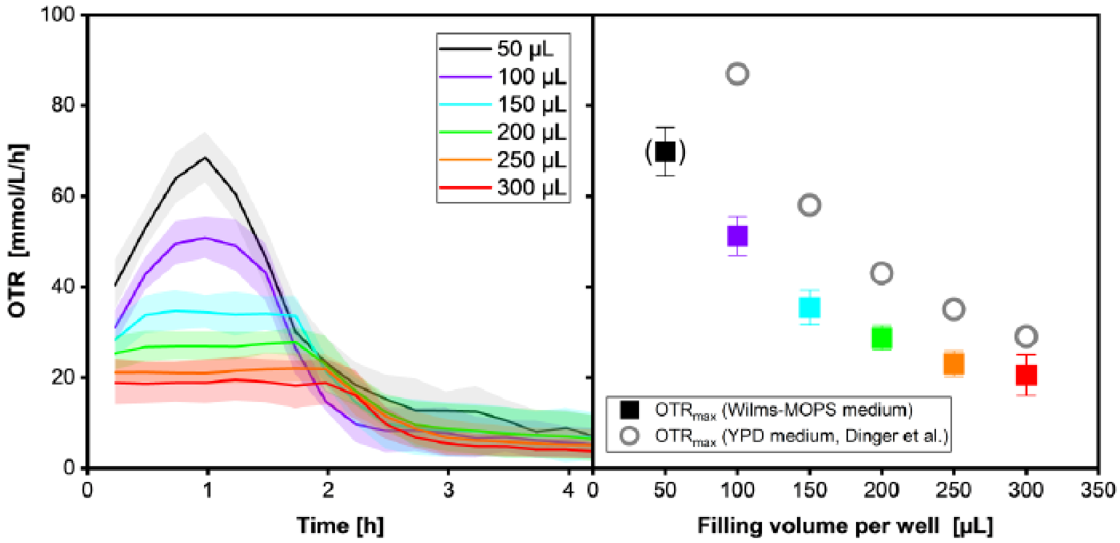

Fig. S5 Empirical OTR_{max} determination *V. natriegens* Vmax pET19b::LevS1417 in Wilms-MOPS medium (20 g/L glucose, 200 mM MOPS). Initial OD_{600} 0.5, 37 °C, variation of the filling volume in 96-DeepWell plate, 1000 rpm at 3 mm shaking diameter. n ≥ 10 replicates per filling volume tested. Since it was unclear whether the maximum oxygen transfer capacity (OTR_{max}) was reached/recorded within the 15-minute measurement intervals during the cultivation with 50 µL filling volume, the value is marked with brackets. The values Dinger et al. (2022) obtained on YPD medium using *Hansenula polymorpha* are marked as grey circles. The difference is due to the medium osmolality, which is much lower for YPD medium.**Table S1 HPLC results for an offline-sampled batch MTP**. n.a.: Not detected. The MTP was inoculated starting from the side that contained the wells with 200 µL filling volume.

Experimental condition		HPLC result					
g/L	µL	g/L	g/L	g/L	g/L	g/L	g/L
Glucose (start)	Filling volume	Glucose	Lactate	Acetate	Succinate	Formiate	Ethanol
0	100	n.a.	n.a.	n.a.	n.a.	n.a.	n.a.
0.5	100	0.50	n.a.	n.a.	n.a.	n.a.	n.a.
1	100	0.97	n.a.	n.a.	n.a.	n.a.	n.a.
1.5	100	1.49	n.a.	n.a.	n.a.	n.a.	n.a.

2	100	2.02	n.a.	n.a.	n.a.	n.a.	n.a.
2.5	100	2.44	n.a.	n.a.	n.a.	n.a.	n.a.
0	100	0.06	n.a.	n.a.	n.a.	n.a.	n.a.
0.5	100	0.50	n.a.	n.a.	n.a.	n.a.	n.a.
1	100	1.05	n.a.	n.a.	n.a.	n.a.	n.a.
1.5	100	1.58	n.a.	n.a.	n.a.	n.a.	n.a.
2	100	1.92	n.a.	n.a.	n.a.	n.a.	n.a.
2.5	100	2.44	n.a.	n.a.	n.a.	n.a.	n.a.
0	100	n.a.	n.a.	n.a.	n.a.	n.a.	n.a.
0.5	100	0.50	n.a.	n.a.	n.a.	n.a.	n.a.
1	100	1.05	n.a.	n.a.	n.a.	n.a.	n.a.
1.5	100	1.53	n.a.	n.a.	n.a.	n.a.	n.a.
2	100	2.06	n.a.	n.a.	n.a.	n.a.	n.a.
2.5	100	2.42	n.a.	n.a.	n.a.	n.a.	n.a.
0	100	n.a.	n.a.	n.a.	n.a.	n.a.	n.a.
0.5	100	0.51	n.a.	n.a.	n.a.	n.a.	n.a.
1	100	1.06	n.a.	n.a.	n.a.	n.a.	n.a.
1.5	100	1.68	n.a.	n.a.	n.a.	n.a.	n.a.
2	100	2.19	n.a.	n.a.	n.a.	n.a.	n.a.
2.5	100	2.37	n.a.	n.a.	n.a.	n.a.	n.a.
3	100	3.07	n.a.	n.a.	n.a.	n.a.	n.a.
3.5	100	3.51	n.a.	n.a.	n.a.	n.a.	n.a.
4	100	4.05	n.a.	n.a.	n.a.	n.a.	n.a.
4.5	100	4.47	n.a.	n.a.	n.a.	n.a.	n.a.
5	100	5.07	n.a.	n.a.	n.a.	n.a.	n.a.
0	100	n.a.	n.a.	n.a.	n.a.	n.a.	n.a.
3	100	3.04	n.a.	n.a.	n.a.	n.a.	n.a.
3.5	100	3.59	n.a.	n.a.	n.a.	n.a.	n.a.
4	100	3.97	n.a.	n.a.	n.a.	n.a.	n.a.
4.5	100	4.19	n.a.	n.a.	n.a.	n.a.	n.a.
5	100	3.39	n.a.	n.a.	n.a.	n.a.	n.a.
0	100	0.97	n.a.	n.a.	n.a.	n.a.	n.a.
3	100	3.06	n.a.	n.a.	n.a.	n.a.	n.a.
3.5	100	3.52	n.a.	n.a.	n.a.	n.a.	n.a.
4	100	4.02	n.a.	n.a.	n.a.	n.a.	n.a.
4.5	100	4.59	n.a.	n.a.	n.a.	n.a.	n.a.
5	100	5.05	n.a.	n.a.	n.a.	n.a.	n.a.
0	100	n.a.	n.a.	n.a.	n.a.	n.a.	n.a.
3	100	3.05	n.a.	n.a.	n.a.	n.a.	n.a.
3.5	100	3.47	n.a.	n.a.	n.a.	n.a.	n.a.
4	100	3.86	n.a.	n.a.	n.a.	n.a.	n.a.
4.5	100	4.03	n.a.	n.a.	n.a.	n.a.	n.a.
5	100	3.37	n.a.	n.a.	n.a.	n.a.	n.a.
0	100	1.06	n.a.	0.22	n.a.	n.a.	n.a.
0	200	n.a.	n.a.	n.a.	n.a.	n.a.	n.a.
0.5	200	n.a.	n.a.	0.22	n.a.	n.a.	n.a.
1	200	0.71	n.a.	0.18	n.a.	n.a.	n.a.
1.5	200	1.20	n.a.	0.17	n.a.	n.a.	n.a.
2	200	0.60	n.a.	0.42	0.12	n.a.	0.18
2.5	200	2.26	n.a.	n.a.	n.a.	n.a.	n.a.
0	200	n.a.	n.a.	n.a.	n.a.	n.a.	n.a.
0.5	200	n.a.	n.a.	0.23	n.a.	n.a.	n.a.
1	200	n.a.	n.a.	0.38	n.a.	n.a.	n.a.
1.5	200	n.a.	n.a.	0.48	0.13	n.a.	0.21
2	200	n.a.	n.a.	0.68	0.20	n.a.	0.32
2.5	200	0.07	n.a.	0.68	0.22	n.a.	0.32
0	200	n.a.	n.a.	n.a.	n.a.	n.a.	n.a.
0.5	200	n.a.	n.a.	0.22	n.a.	n.a.	n.a.
1	200	n.a.	n.a.	0.34	n.a.	n.a.	n.a.
1.5	200	n.a.	n.a.	0.45	0.12	n.a.	0.18
2	200	0.08	n.a.	0.54	0.15	n.a.	0.25
2.5	200	n.a.	n.a.	0.68	0.20	n.a.	0.33
0	200	n.a.	n.a.	n.a.	n.a.	n.a.	n.a.
0.5	200	n.a.	n.a.	0.23	n.a.	n.a.	n.a.
1	200	n.a.	n.a.	0.37	0.11	n.a.	n.a.
1.5	200	n.a.	n.a.	0.56	0.17	n.a.	0.24
2	200	n.a.	n.a.	0.78	0.30	n.a.	0.35
2.5	200	n.a.	n.a.	0.80	0.33	n.a.	0.34
3	200	n.a.	n.a.	0.79	0.26	n.a.	0.38

3.5	200	n.a.	n.a.	0.90	0.30	n.a.	0.45
4	200	n.a.	n.a.	1.10	0.43	2.86	0.53
4.5	200	2.71	n.a.	0.52	0.17	n.a.	0.23
5	200	n.a.	n.a.	1.25	0.49	n.a.	0.68
0	200	n.a.	n.a.	n.a.	n.a.	n.a.	n.a.
3	200	n.a.	n.a.	0.84	0.28	n.a.	0.35
3.5	200	n.a.	n.a.	0.98	0.37	n.a.	0.44
4	200	n.a.	n.a.	1.08	0.46	n.a.	0.50
4.5	200	n.a.	n.a.	1.08	0.43	n.a.	0.55
5	200	n.a.	n.a.	1.09	0.41	n.a.	0.54
0	200	n.a.	n.a.	n.a.	n.a.	n.a.	n.a.
3	200	n.a.	n.a.	0.85	0.33	n.a.	0.38
3.5	200	n.a.	n.a.	1.02	0.39	n.a.	0.48
4	200	n.a.	n.a.	1.12	0.43	4.10	0.55
4.5	200	n.a.	n.a.	1.23	0.56	n.a.	0.59
5	200	n.a.	n.a.	1.36	0.53	n.a.	0.67
0	200	n.a.	n.a.	n.a.	n.a.	n.a.	n.a.
3	200	n.a.	n.a.	0.88	0.33	n.a.	0.36
3.5	200	n.a.	n.a.	1.00	0.41	n.a.	0.45
4	200	n.a.	n.a.	1.15	0.45	n.a.	0.56
4.5	200	n.a.	n.a.	1.26	0.51	n.a.	0.61
5	200	n.a.	n.a.	1.25	0.45	n.a.	0.65
0	200	n.a.	n.a.	n.a.	n.a.	n.a.	n.a.

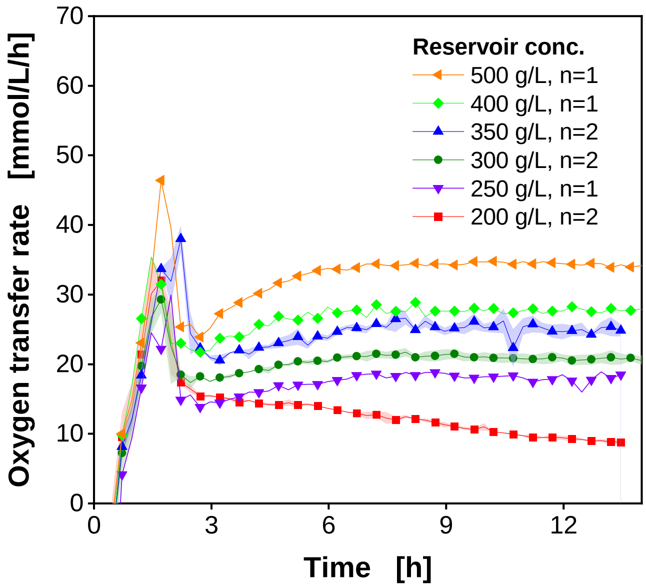

Fig. S6 Membrane-based fed-batch cultivation in shake flasks at different glucose concentrations in the feed reservoir *V. natriegens* Vmax pET19b::inuGB-V3 in modified Wilms-MOPS medium (no initial glucose). 10 mL filling volume in 250 mL RAMOS flasks, initial OD_{600} 0.5, 37 °C, 350 rpm at 50 mm shaking diameter. The oxygen transfer rate was monitored using a RAMOS device. Oxygen transfer rate over time for different glucose concentrations in the reservoir. For clarity, only every third data point is indicated by a symbol. Shadows indicate minimum/maximum for n = 2.

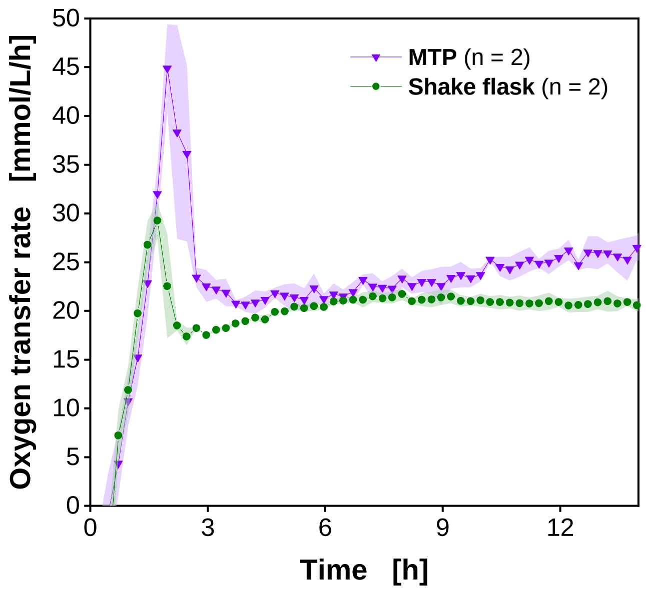

Fig. S7 Comparison between fed-batch in microtiter plate and in shake flask. *V. natriegens* Vmax pET19b::inuGB-V3 in modified Wilms-MOPS medium (no initial glucose), initial OD_{600} 0.5, 37 °C. Microtiter plate (MTP): 100 µL filling volume in untreated 96-Well FeedPlate (SMFP04001, high release), 1000 rpm at 3 mm shaking diameter. The oxygen transfer rate was monitored using a µTOM device. Shake flask: 10 mL filling volume in 250 mL RAMOS flasks, 300 g/L glucose concentration in reservoir, 350 rpm at 50 mm shaking diameter. The oxygen transfer rate was monitored using a RAMOS device. The mean value is shown. The shadows indicate minimum/maximum for n = 2 replicates.

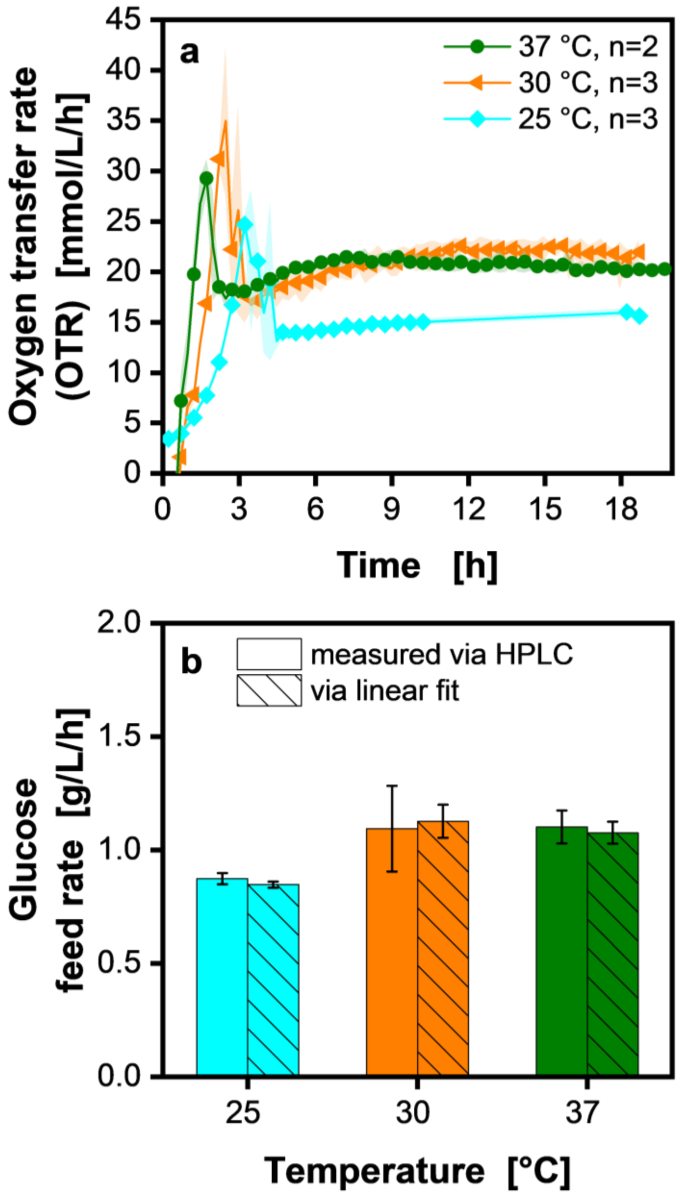

Fig. S8 Influence of the cultivation temperature on the membrane-based fed-batch cultivation in shake flasks Non-induced fed-batch cultivation of *V. natriegens* Vmax pET19b::inuGB-V3 in modified Wilms-MOPS medium (no initial glucose, 300 g/L glucose in feed reservoir) with 400 mM MOPS buffer. 10 mL filling volume in 250 mL RAMOS flasks, initial OD_{600} 0.5, 350 rpm at 50 mm shaking diameter. The oxygen transfer rate was monitored using a RAMOS device. Comparison of fed-batch processes at 37 °C, 30 °C, and 25 °C. (a) Oxygen transfer rate over time. For clarity, only every third data point is shown as a symbol. Shadows indicate the standard deviation for n = 3 or the minimum/maximum for n = 2. (b) Glucose feed rates depending on the cultivation temperature: The feed rate was either offline measured through HPLC analysis (open bars) or calculated via the linear fit presented in Fig. 2b (hatched bars).

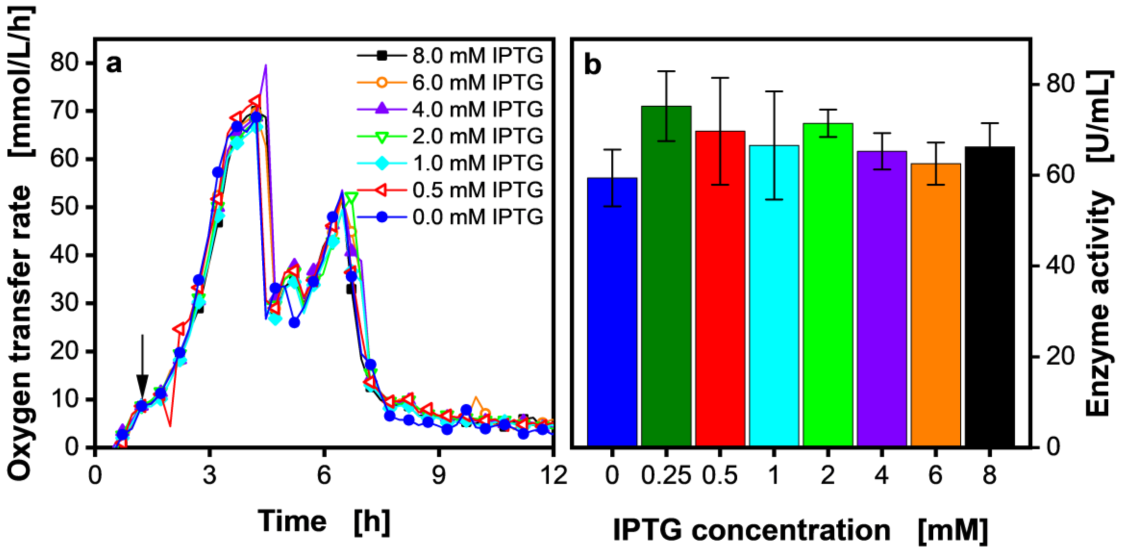

Fig. S9 Influence of the inducer concentration on inulosucrase production in a batch process *V. natriegens* Vmax pET19b::inuGB-V3 in modified Wilms-MOPS medium (20 g/L glucose). 8 mL filling volume in 250 mL RAMOS flasks, initial OD_{600} 0.5, 30 °C, 350 rpm at 50 mm shaking diameter. The oxygen transfer rate was monitored using a RAMOS device. Induction using 0 - 8 mM IPTG at OD_{600} 1. **(a)** Oxygen transfer rate over time, arrow indicates induction time. For clarity, only every third data point is indicated by a symbol. **(b)** Inulosucrase activity after 19 h. For the cultivations induced with 0, 0.25, and 0.5 mM IPTG, a standard ANOVA showed no significant difference between the data of Fig. 5 and Fig. S7. Homogeneity of variance was tested using Levene's test with squared deviations.

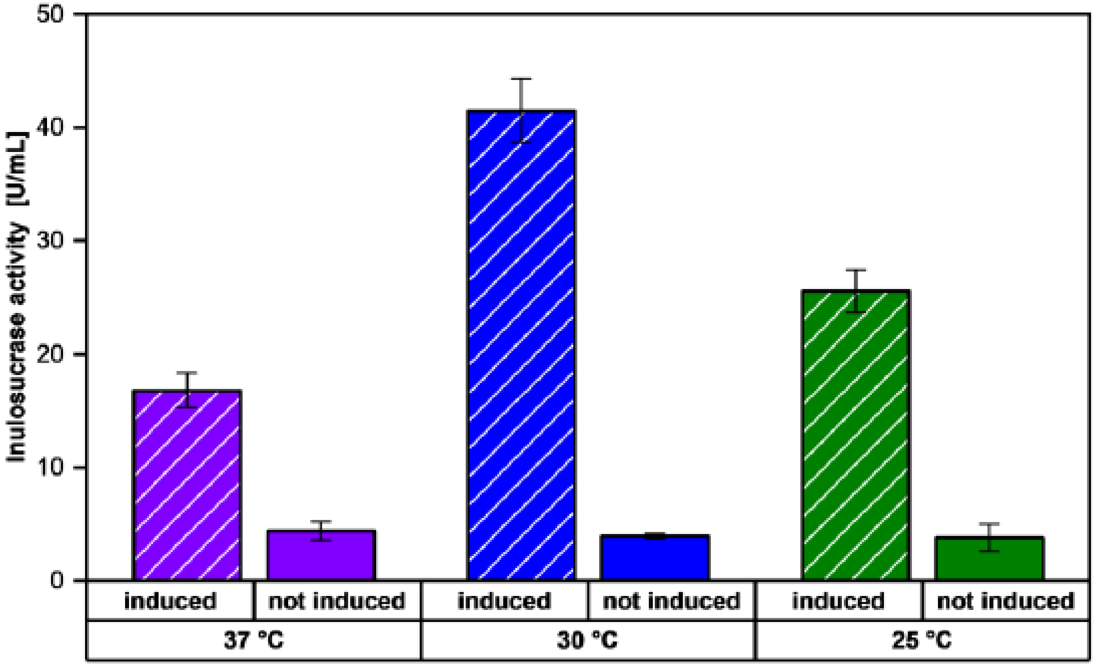

Fig. S10 Offline results for expression of the inulosucrase InuGB-V3 in *E. coli* at different temperatures *E. coli* BL21 (DE3) pET19b(+)::inuGB-V3 in Wilms-MOPS medium (20 g/L glucose). Initial OD_{600} 0.5, 8 mL in 250 mL RAMOS flask, 350 rpm at 50 mm shaking diameter. Induced: Induction at OD_{600} 1.5-1.6 with 0.025 mM IPTG by adding 100 µL of a correspondingly higher concentrated IPTG solution. Not induced: Addition of 100 µL of DI-water at OD_{600} 1.5-1.6.

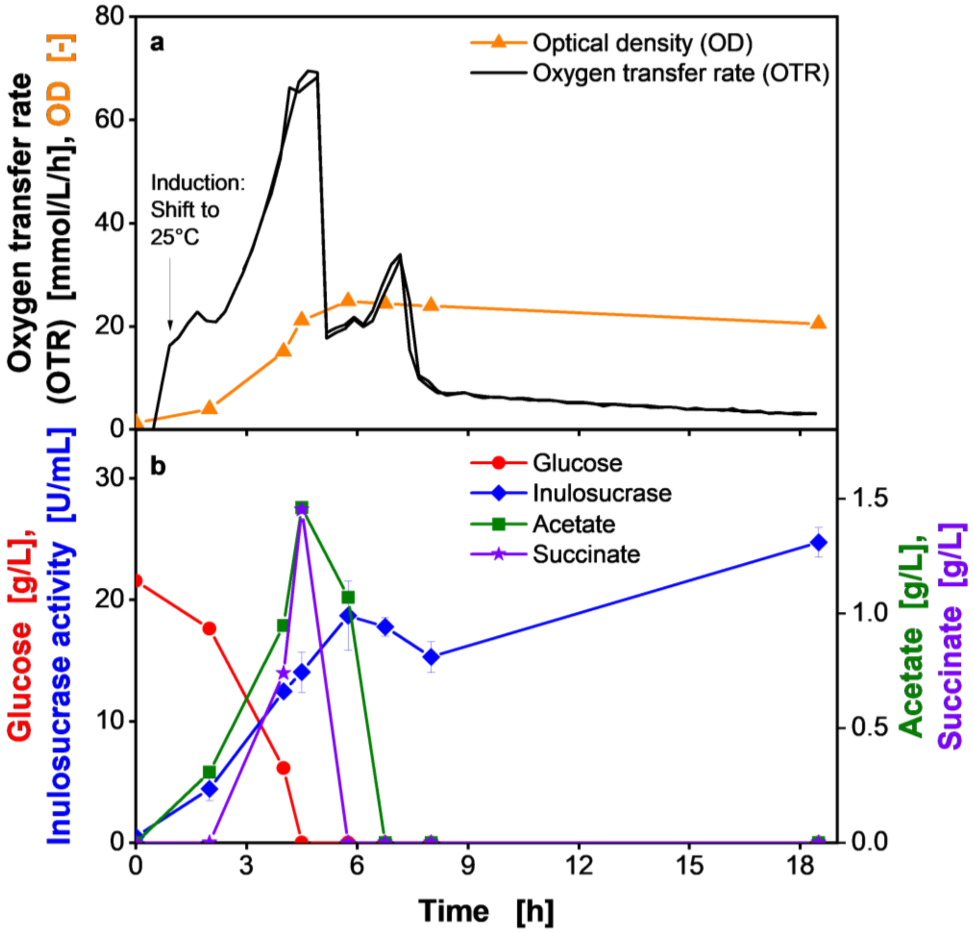

Fig. S11 Sampling during a batch process *V. natriegens* Vmax pET19b::inuGB-V3 in modified Wilms-MOPS medium (20 g/L glucose). 8 mL filling volume in 250 mL RAMOS flasks, initial OD_{600} 0.5, 350 rpm at 50 mm shaking diameter. The oxygen transfer rate was monitored using a RAMOS device. Cultivation at 37°C, induction using 0.025 mM IPTG at OD 1, and expression at 25°C after induction. (a) Oxygen transfer rate (OTR) over time (black), two replicates, arrow indicates the induction time. Optical density (OD) in orange. (b) Glucose concentration (red), acetate concentration (green), and succinate concentration (violet) determined via HPLC, inulosucrase activity (blue).

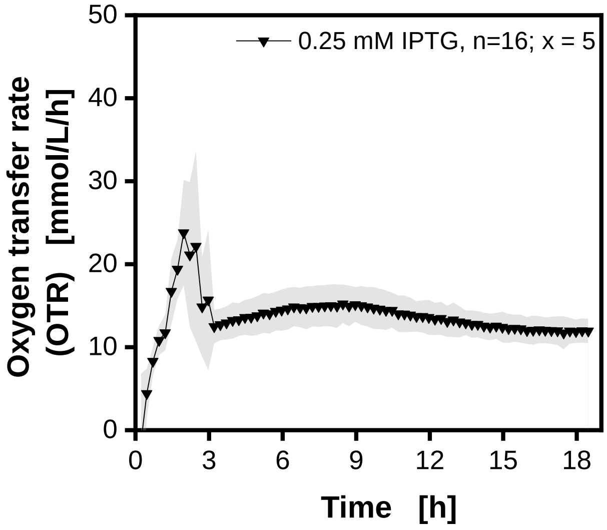

Fig. S12 Repeatability of the fed-batch fermentation in membrane-based shake flasks Fed-batch cultivation of *V. natriegens* Vmax pET19b::inuGB-V3 in modified Wilms-MOPS medium (no initial glucose). Induction with 0.25 mM IPTG at OD_{600} 1.5. 10 mL filling volume in 250 mL RAMOS flasks, 300 g/L glucose concentration in feed reservoir, initial OD_{600} 0.5, 30 °C, 350 rpm at 50 mm shaking diameter. Shown are n = 16 replicates from x = 5 different experiments.

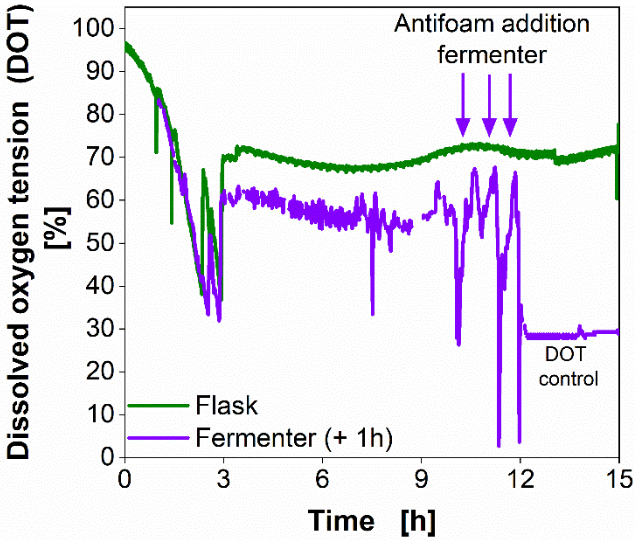

Fig. S13 Dissolved oxygen tension (DOT) of fed-batch fermentations in the membrane-based fed-batch shake flask (green) and a bench-top fermenter (purple). Fed-batch cultivation of *V. natriegens* Vmax pET19b::inuGB-V3 in modified Wilms-MOPS medium. Shake flask: 10 mL filling volume in 250 mL RAMOS flasks, 300 g/L glucose concentration in feed reservoir, initial OD_{600} 0.5, 30 °C, 350 rpm at 50 mm shaking diameter**.** Fermenter**:** 1 L filling volume, 0.21 L feed solution, 117 g/L glucose with a feed rate of 17.40 mg/h, 30 °C, 700 rpm and 0.5 vvm. Cable ties were attached to the agitator shaft as a mechanical foam destroyer. The purple arrows indicate antifoam addition into the bench top fermenter. Due to heavy foaming, the DOT set point of 30 % was reached after 12 hours.

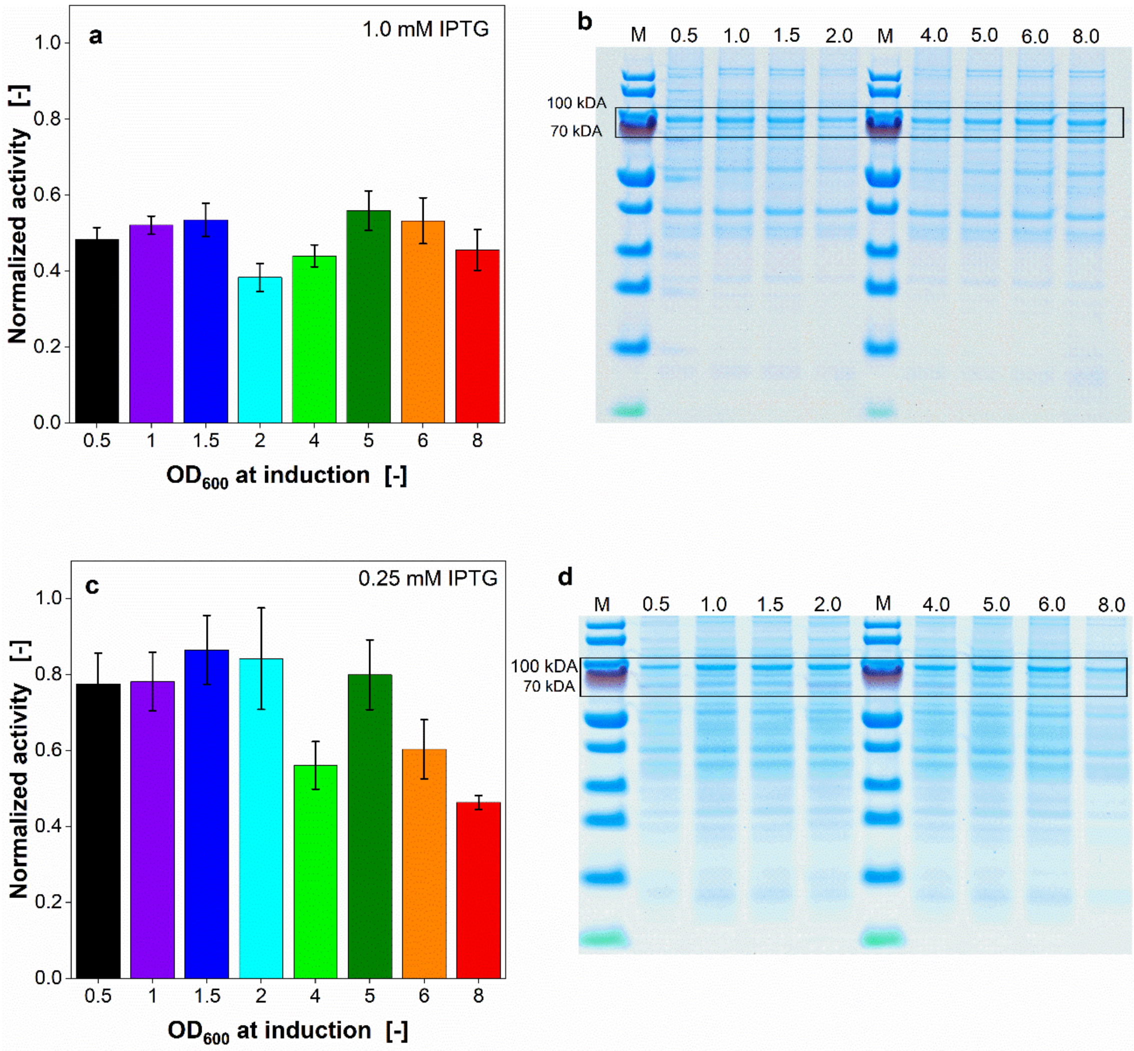

Fig. S14 Normalized inulosucrase activity of induced batch cultivations of *V. natriegens* in comparison to SDS-PAGE. (a) Induction with 1 mM IPTG at different OD_{600}. (b) SDS-PAGE of the cultivations with 1 mM of IPTG induction. The numbers represent the OD_{600} at the time of induction. M represents the Marker. (c) Induction with 0.25 mM IPTG at different OD_{600}. (d) SDS-PAGE of the cultivations with 0.25 mM of IPTG induction. The numbers represent the OD_{600} at the time of induction. M represents the marker. The box on the gels highlights the band where the target protein is expected.

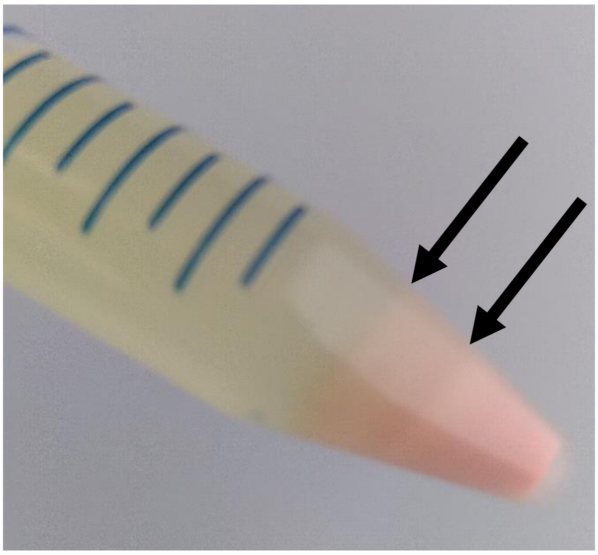

Fig. S15 Exopolysaccharide formation becomes visible as a third phase after centrifugation of cell broth (corresponds to the experiment in Fig. 8) Fed-batch cultivation of *V. natriegens* Vmax pET19b::inuGB-V3 in modified Wilms-MOPS medium (no initial glucose). Induction with 0.25 mM IPTG at OD_{600} 1.5. 10 mL filling volume in 250 mL RAMOS flasks, 300 g/L glucose concentration in feed reservoir, initial OD_{600} 0.5, 37 °C, 350 rpm at 50 mm shaking diameter. Arrows indicate phase boundaries of cell broth and cell pellet; exopolysaccharide emulsion is visible between the arrows.
